# Tumor suppressor RARRES1- A novel regulator of fatty acid metabolism in epithelial cells

**DOI:** 10.1371/journal.pone.0208756

**Published:** 2018-12-17

**Authors:** Sara Maimouni, Naiem Issa, Selina Cheng, Chokri Ouaari, Amrita Cheema, Deepak Kumar, Stephen Byers

**Affiliations:** 1 Department of Biochemical, Molecular and Cellular Biology, Georgetown University, Washington, District of Columbia, United States of America; 2 Georgetown-Lombardi Comprehensive Cancer Center, Department of Oncology, Georgetown University, Washington, District of Columbia, United States of America; 3 University of the District of Columbia, Washington, District of Columbia, United States of America; 4 Julius L. Chambers Biomedical Biotechnology Research Institute, North Carolina Central University, Durham, North Carolina, United States of America; University of South Alabama, UNITED STATES

## Abstract

Retinoic acid receptor responder 1 (RARRES1) is silenced in many cancers and is differentially expressed in metabolism associated diseases, such as hepatic steatosis, hyperinsulinemia and obesity. Here we report a novel function of RARRES1 in metabolic reprogramming of epithelial cells. Using non-targeted LC-MS, we discovered that RARRES1 depletion in epithelial cells caused a global increase in lipid synthesis. RARRES1-depleted cells rewire glucose metabolism by switching from aerobic glycolysis to glucose-dependent *de novo* lipogenesis (DNL). Treatment with fatty acid synthase (FASN) inhibitor, C75, reversed the effects of RARRES1 depletion. The increased DNL in RARRES1-depleted normal breast and prostate epithelial cells proved advantageous to the cells during starvation, as the increase in fatty acid availability lead to more oxidized fatty acids (FAO), which were used for mitochondrial respiration. Expression of RARRES1 in several common solid tumors is also contextually correlated with expression of fatty acid metabolism genes and fatty acid-regulated transcription factors. Pathway enrichment analysis led us to determine that RARRES1 is regulated by peroxisome proliferating activated receptor (PPAR) signaling. These findings open up a new avenue for metabolic reprogramming and identify RARRES1 as a potential target for cancers and other diseases with impaired fatty acid metabolism.

## Introduction

Retinoic acid receptor responder element 1 (RARRES1), also known as tazarotene induced gene 1 (TIG1), was initially identified as a novel retinoic acid receptor-regulated gene in the skin [1]. We showed in prostate cancer cells that RARRES1 is able to induce autophagy, decrease mechanistic target of rapamycin (mTOR) and increase Sirtuin 1 (SIRT1), two important regulators of energy homeostasis [2]. RARRES1 also induces autophagy in cervical cancer cells [3]. The ability of RARRES1 to regulate the expression of metabolic master regulators and induce autophagy suggests that it might function to reprogram metabolism in cells.

RARRES1 is differentially expressed in metabolic diseases and is associated with biological hallmarks that require metabolic reprogramming. For example, RARRES1 is among the most up-regulated genes in subcutaneous fat from obese human subjects on a diet-induced weight loss and among the most downregulated genes during weight maintenance [4]. In adipocyte differentiation, in which metabolic reprogramming is crucial, RARRES1 is increased during dedifferentiation and decreased during differentiation [5]. RARRES1 is also differentially expressed in mouse models of hepatic steatosis and cholestatic liver disease [6,7].

Although RARRES1 is among the most commonly methylated genes in multiple cancers, it is actually increased in basal-like hormone receptor negative breast cancer and in liver cirrhosis, a risk factor for hepatocellular cancer [8–10]. Metabolic reprogramming is now considered a hallmark of cancer etiology [11]. For decades, aerobic glycolysis (Warburg Effect) was considered to be the most important energetic pathway that cancer cells use to survive and proliferate[12]. However, it is now clear that oxidative phosphorylation and fatty acid metabolism play a major role in cancer progression and drug resistance [13].

Here we demonstrate that RARRES1 is regulated by starvation and that its depletion in epithelial cells reprograms metabolism by modulating a switch from aerobic glycolysis to glucose dependent *de novo* lipogenesis (DNL). RARRES1 depletion modulated DNL and increased substrates (endogenous fatty acids) for fatty acid oxidation during serum starvation and pharmacological inhibition of fatty acid synthase. We also show that *RARRES1* expression correlates with that of fatty acid metabolism genes in breast, colorectal and prostate cancers. Two of these genes, peroxisome proliferating activated receptor alpha and gamma (PPARα and PPARγ) were found to regulate RARRES1 expression in epithelial cells. These findings identify RARRES1 as a novel modulator of lipid metabolism.

## Materials and methods

### Cell-culture

HEK293T was cultured in DMEM (Gibco-Invitrogen, Grand Island, NY) supplemented with 5% fetal bovine serum. The cells were maintained in a humidified modular incubator at 37° C and 5% CO_2_. MCF 10A cells were maintained in DMEM/F12 (50:50 mix) supplemented with 5% horse serum, 10 mM HEPES, 10 ug/ml insulin, 20 ng/ml epidermal growth factor, 0.5 ug/ml hydrocortisone, 100 ng/ml cholera toxin. Immortalized human prostate epithelial PWR-1E cells (a gift from Dr. S.C. Chauhan, University of South Dakota) were maintained according to ATCC’s recommendation. The passage numbers of all cell lines were below 30 between thawing and use in the experiments. All cell lines were authenticated and tested for mycoplasma by the Lombardi Comprehensive Cancer Center Tissue Culture Shared Resources.

### Antibodies and reagents

Primary antibodies targeting the following antigens were used: rabbit anti-human RARRES1 (TIG1) (Sigma-Aldrich, St. Louis, MO), GAPDH (Fitzgerald Industries International, Acton, MA) and alpha-tubulin (Sigma-Aldrich, St. Louis, MO). C75 compound was obtained from Cayman Chemicals. Oleic acid was purchased from Cayman Chemicals.

### Cell extracts

Cells were washed with PBS and lysed in RIPA lysis buffer (1% sodium deoxycholate, 0.1% SDS, 1% Triton X-100, 10mM Tris-HCL pH8, 150 mM NaCl) for 15 minutes at 4°C. Protein concentration was determined using a protein microplate assay (Biorad Laboratories).

### RNA extraction and real-time quantitative PCR Analysis

Total RNA was isolated from cells by using Trizol reagent (Invitrogen) according to the instructions of the manufacturer. RNA was isolated using the RNeasy kit (Qiagen) according to the instructions of the manufacturer. Reverse transcription was done using Invitrogen Reverse Transcription Kit. The cDNA samples were used to quantify RARRES1 expression using the fast real-time PCR kit (Thermo Fisher) with the appropriate TaqMan probes (Thermo Fisher: RARRES1 Hs00894859_m1 and 18s Hs03003631_g1) in a StepOne (Applied Biosystems) compact qPCR machine. Transcript levels were normalized to the level of 18S.

### Plasmid and siRNA constructs

RARRES1 were directionally cloned into the *Bgl*II and *Hin*dIII sites in the pEYFP-N1 vector (Clontech). Full-length RARRES1 (+1 - +897) were cloned into the pGlue vector as codon optimized versions by Genscript (Piscataway, NJ). RARRES1 (cat#M-012937-00) and non-targeting control siRNAs (cat#D-001210-01-20) were from Dharmacon (Lafayette, CO). Plasmid DNA was introduced by lipofectamine 3000 and siRNA constructs were transfected using RNAi Max. Constructs for stable depletion of RARRES1 were obtained from the RNAi Consortium (Moffat et al., 2006) via SIGMA-Aldrich (NM_002888). For the RARRES1 gene, five pre-made constructs were obtained and individually tested to identify those able to achieve efficient knockdown at the protein level. Negative control constructs in the same vector system (vector alone, pLKO.1 puro) were obtained from SIGMA-Aldrich (NM_003177). Next, MCF10A cells were infected with shRNA lentiviruses. To do this, the cells were plated at sub confluent densities. The next day, the cells were infected with a cocktail of 100 ul virus-containing medium, 1 ml regular medium, and 8 μg/ml Polybrene. The medium was changed 1-day post-infection, and selection medium was added 2 days post-infection (5 μg/ml puromycin for MCF10A). After 3 days of puromycin selection, the mock-infected cells had all died. Stably infected pooled clones were studied.

### Oleic acid treatment and nutrient deprivation

Oleic acid treatment was used as recommended by Cayman Chemicals; MCF 10A and HEK 293 T cells were treated at a 1:5000 dilution for 4 hours or overnight respectively. MCF 10A cells were plated and grown in serum rich DMEM media that was depleted of serum for 18, 24 or 40 hours. Cells were then harvested for qPCR or western blot analysis.

### Western blot analysis

To confirm RARRES1-YFP expression and RARRES1 knockdown in MCF 10A or HEK293 T cells and RARRES1 overexpression in serum starved cells, samples were subjected to western blot analysis (**S4D** and **S8 Figs**). 50 μg of cell lysate was separated on 4–12% SDS/PAGE, transferred to nitrocellulose membrane (Amersham Pharmacia Biotech), blocked with 5% milk-PBS, and incubated in primary antibody in 5% milk-PBS overnight at 4°C. Blots were washed 3 times for 5 minutes each in PBS-0.5% tween, followed by incubation in HRP-conjugated secondary antibody (KPL, Gaithersberg, MD) for 1 hour at room temperature on an orbital. Blots were washed 2 times in PBS-0.5% tween, followed by 1 wash in PBS. Detection of immunoreactive bands was carried out using chemiluminescence with ECL Western Blotting Detection Reagents (Amersham, Piscataway, NJ). Band intensities were quantified using ImageJ [14].

### Docosahexaenoic acid treatment

761 mM DHA stock was diluted to 1 mg/mL in 1XPBS. The DHA working solution was incubated for 1 hour at 37°C. The solution was then diluted in the cells’ media to the desired concentration. The cells were incubated for 30 min, 1 hour, 2 hours or overnight (17 hours) at a concentration of 50 μM or 200 μM. DHA was purchased from Cayman Chemicals (Item #90310) or vehicle (95% ethanol (EtOH)). After incubation time, the cells were then harvested in TRIzol, purchased from Thermo Fisher Scientific (Cat# 15596026).

### Nile Red lipophilic staining and Oil Red O staining

Following RARRES1 or scrambled siRNA transfection, MCF 10A cells were fixed with 10% formaldehyde (Protocols) for 15 minutes at room temperature and washed 3 times with 1X PBS. To stain with Nile Red (Sigma, St. Louis, MO, USA), 1 μL of a 1 mg/mL stock solution was added to 10 mL of 150 mM NaCl in PBS to make a Nile Red solution. The Nile Red solution was added to the cells and incubated for 10 minutes in the dark. Cells were counterstained with DAPI. Cells were imaged with the Keyence BZ-X microscope (Keyence Corporation, Osaka, Japan).

MCF 10A cells transfected with siRNA were grown for 48 hours. HEK 293 T cells and MCF 10A cells transfected with YFP or RARRES1-YFP plasmids were grown for 24 hours then treated with oleic acid for 4 hours or overnight before fixation. Cells were fixed in 10% formalin (Electron Microscopy Science, Hatfield, PA), for 10 min. at room temperature. Cells were stained for neutral lipids using the oil red O staining kit (American Master Tech Scientific, Lodi, CA). Nile Red was not used in this case because the most accurate way to detect the lipids via Nile Red staining is through the green channel, the same channel is used to detect YFP [15,16]. Briefly, cells were washed four times with ddH20 (EMD Chemicals Inc., San Diego, CA), incubated for 10 min RT with oil red O, and counterstained with DAPI (Thermo-Scientific). Cells were imaged with the Keyence BZ-X microscope. For both staining methods, oleic acid treatment was used as a positive control. Intensity of lipid staining was quantified through ImageJ [14].

### Metabolic analysis using extracellular flux assays

Bioenergetics profile of transient RARRES1 knockdown in MCF10A and PWR-1E cells and stable RARRES1 knockdown MCF10A cells were measured using the XF^e^96 Extracellular Flux Analyzer. Oxygen consumption rates and response to mitochondrial stress factors were analyzed by using the XF Cell Mito Stress Kit. 48 hours prior to analysis, MCF10A and PWR-1E cells were transfected with RARRES1 siRNA or scramble siRNA in duplicates. The night before the assay, the cells were seeded at an optimized cell density (MCF10A cells: 10,000 and PWR-1E cells: 20,000) in the 96-well XF^e^ plate and incubated at 37°C with 5% CO2 overnight. The day of analysis, the cells were incubated at 37°C in a CO_2_-free atmosphere incubator for 1 hour. Basal oxygen consumption rate and extracellular acidification rate were measured. Subsequently, oxygen consumption rate (OCR) and extracellular acidification rate (ECAR) responses were observed after separate injections of oligomycin (1 μM), carbonyl cyanide-p-trifluoromethoxyphenylhydrazone FCCP (0.5 μM for MCF10A cells or 0.25 μM for PWR-1E cells), and a combination of rotenone and antimycin A (0.5 μM) were respectively prompted in the assay. For each injection, there was a total of 3 cycles, each one lasted 3 minutes and a measurement was taken at the beginning of each cycle. Glycolysis was also analyzed, using the XF Glycolysis Stress Test kit, in the XFe96 Extracellular Flux Analyzer. Cells were transiently knocked down with RARRES1 siRNA or scramble negative control siRNA and plated at the same optimal density as the XF Cell Mito Stress Test protocol. ECAR was measured at baseline and after adding glucose (10 mM), oligomycin (1 μM) and 2-deoxy-d-glucose (100 mM) respectively. Fatty Acid Oxidation measurements were done as recommended by Agilent Seahorse. Cells in nutrient-rich media, were treated with BPTES (a glutaminolysis inhibitor), UK-5099 (a glycolysis inhibitor) and etomoxir (a fatty acid oxidation inhibitor). The cells were assessed for their dependency on each pathway and their plasticity when each pathway is inhibited. To measure fatty acid oxidation in a starved state, cells were starved with minimal substrate DMEM for 24 hours. The minimal substrate media included 1% serum, 1 mM glutamine, 0.5 mM carnitine, and 0.5 mM of glucose. Insulin, EGF and hydrocortisone were excluded from the media. The day of the assay, starved cells were washed and incubated with 1X KHB (111 mM NaCl, 4.7 mM KCl, 1.25 mM CaCl2, 2 mM MgSO4, 1.2 mM NaH2PO4 and supplemented with 2.5 mM glucose, 0.5 mM carnitine, and 5 mM) in a non-CO2 37°C incubator. 15 minutes prior to the assay, 40 μM etomoxir was added to the cells to measure endogenous fatty acid uptake for FAO. To measure exogenous fatty acid uptake for FAO, Palmitate-BSA and BSA (vehicle) were added to cells right before treating with etomoxir and adding the cells to the Flux Analyzer. All experiments were analyzed in duplicates for at least two independent experiments for each cell line and the results were normalized to their protein concentration or cell number.

### ATP Detection

Cells were transfected with RARRES1 siRNA or scrambled siRNA by RNAiMax in a 6-well plate. These cells were left to grow for 24 hours and subsequently replated in a 96-well plate. The cells were then treated with 150 μM of C75 for 2, 3 and 5 hours. The cells were then harvested, and ATP was quantified by luminescence according to the manufacturer’s instructions (Cayman Chemicals, Ann Arbor, MI, USA). ATP standards were included (0.01 μM to 10 μM).

### LC-MS and GC-MS metabolite extraction

Cell samples were processed using 150 μL 100% water. Samples were plunged in to dry ice for 30 seconds and heat shocked in a water bath for 90 sec. at 37 ⁰C. Protein quantification was done using the Bradford Assay. The remainder of the sample was processed using 600 μL of methanol containing internal standard. Chloroform was added to each sample (600 μL) and centrifuged (13,000 rpm) at 4⁰C for 20 min. Chilled Acetonitrile (600 μL) was added to each phase and allowed to incubate at -20 ⁰C to further precipitate cellular debris and proteins. Incubated samples were centrifuged (13,000 rpm) at 4⁰C for 20 min. Supernatants from the incubated samples were transferred to another Eppendorf tube, dried under vacuum, and stored at -80°C until analysis. For analysis, the dried samples were reconstituted in 100 μL of 50% Methanol in water, and both phases combined in a Mass Spec Sample tube for LCMS or GCMS analysis.

### Ultra-performance liquid chromatography-time of flight mass spectrometry (UPLC TOF MS) mm based metabolomic analyses

Each sample (2 L) was injected onto a reverse-phase 50 × 2.1 BEH 1.7 m C18 column using an Acquity UPLC system (Waters Corporation, USA). The gradient mobile phase comprised of: solvent A- 100% water + 0.1% formic acid; solvent B- 100% acetonitrile + 0.1% formic acid; solvent D- 90% isopropanol and 10% acetonitrile + 0.1% formic acid (all containing 0.1% Formic Acid). Each sample was resolved for 13 min at a flow rate of 0.4 mL/min. The gradient consisted of 95% A and 5% B for .50 minutes, then a ramp of curve 6 to 2% A and 98% B from 0.5 min. to 8.0 min., then a ramp of curve 6 to 2% B and 98% D to 9.0 min., a hold of 2% B and 98% D up to 10.5 min., then a ramp of curve 6 to 50% A and 50% B to 11.5 min., then a ramp of curve 6 to 95% A and 5% B to 12.5 min., and a hold of 95% A and 5% B to 13.0 min. The column eluent was introduced directly into the mass spectrometer by electrospray. Mass spectrometry was performed on a quadrupole-time-of-flight mass spectrometer operating in either negative or positive electrospray ionization mode. Positive mode has a capillary voltage of 3.0 kV, a sampling cone voltage of 30 V, and a source offset of 80 V. Negative mode has a capillary voltage of 2.75 kV, a sampling cone voltage of 20 V, and a source offset of 80 V. The de-solvation gas flow was 600 L/hr. and the temperature was set to 500⁰C. The cone gas flow was 25 L/h, and the source temperature was 100⁰C. The data were acquired in the Sensitivity and MS Mode with a scan time of 0.1 seconds, and inter-scan delay at 0.08 seconds. Accurate mass was maintained by infusing Leucine Enkephalin (556.2771 m/z) in 50% aqueous acetonitrile (1.0 ng/mL) at a rate of 10 μL/min via the lock-spray interface every 10 seconds. Data were acquired in centroid mode from 50–1200 m/z mass range for TOF-MS scanning.

### Data pre-processing and metabolite identification and validation

Centroided and integrated UPLC-TOFMS data were pre-processed using the XCMS software normalized to the ion intensity of respective internal standards as well as to the total protein concentration. The data were log transformed and multivariate data analyses were performed to delineate significantly altered metabolites in the two groups. These metabolites were putatively identified via accurate mass-based search using the Madison Metabolomics Consortium Database (MMCD), SimLipid (Premier Biosoft), the Human Metabolome Database (HMDB) and LIPID MAPS [17–19]. Selected metabolites in transient RARRES1 KD and scrambled siRNA cells were validated using tandem mass spectrometry. The daughter and parent ions for the metabolites were matched with the MS/MS spectra available in HMDB, SimLipid and LIPID MAPS (**S2A Fig**). MS^E^ results were also subjected to additional lipid validations through SimLipid software V6.01 (Premier Biosoft, Palo Alto, CA, USA) (**S2B Fig**). This validation method has been reported by others[20,21]. It is important to note that our non-targeted LC-MS method and validation approach did not allow for the identification of the specific lipid that corresponds to each ion, but we were able to identify the lipid classes. Citrate was confirmed by comparing the retention time under the same chromatographic conditions and by matching the fragmentation pattern of the parent ion from the biological sample to that of the standard metabolite using tandem mass spectrometry (UPLC-TOFMS/MS) (**S5 Fig**).

### LC-mass spectrometry statistical analyses

Raw metabolomics data was analyzed using analysis functionalities of Metaboanalyst 4.0 [22]. Data were log transformed before performing t- statistics to identify significant metabolites. Metabolites with an adjusted p-value of less than 0.05 and a fold change either less than or equal to 0.5 or greater than 1.5 were used to create volcano and Partial Least Squares–Discriminant Analysis (PLS-DA) plots (**S1 Fig**).

### GC-TOF single injections

Processed samples were transferred (100 L) to a GC vial with a salinized insert, dried in by speed-vac, caped and kept at 20 degrees for derivatization. The dry residue was dissolved in 15 L of 20.0 mg/mL O-Methoxyamine-Hydrochloride in pyridine. N-Methyl-N-(trimethylsilyl) trifluoroacetamide: MSTFA/BSTFA WITH 1% TMCS (50:50) was added (60 L) and the sample heated 40°C with constant agitation for 30 min. The sample was then allowed to cool to 20°C and incubated for 4 hrs. Before injection for GC-TOF analysis After TMS derivatization, A 5.0 L aliquot of the derivatized solution was injected in (1:5) split mode into an Agilent 7890B GC system (Santa Clara, CA, USA) that was coupled with a Pegasus HT TOF-MS (LECO Corporation, St. Joseph, MI, USA). Separation was achieved on an Rtx-5 w/Integra-Guard capillary column (30 m x 0.25 mm ID, 0.25 m film thickness; Restek Corporation, Bellefonte, PA, USA), with helium as the carrier gas at a constant flow rate of 1.0 mL/min. The temperature of injection, transfer interface, and ion source was set to 150, 270, and 230°C, respectively. Electron impact ionization (70 eV) at full scan mode (*m*/*z* 40–600) was used, with an acquisition rate of 30 spectra per second in the TOF/MS setting.

### GC-MS analysis and metabolite validation

Raw data files were pre-processed through Leco Statistical Compare software to generate an excel output. Output data was normalized by internal standard (4- Nitrobenzoic acid). In Metaboanalyst 4.0 [22], the data was further normalized by log transformation. For data analysis, a T-Test and fold change was performed to generate a volcano plot. Peak detection with background subtraction and subsequent matching of the resulting mass spectra to the Agilent Fiehn GC/MS Metabolomics RTL Library using NIST MS searches was done (**S3 Fig**). The LC-MS and GC-MS raw data are accessible in Dryad: doi:10.5061/dryad.6t1m4k4.

### Bioinformatics analysis

Oncomine^TM^ (Compendia Bioscience, Ann Arbor, MI, USA) was used to identify cancers where *RARRES1* gene expression was significantly upregulated or downregulated. These cancers included breast, cervical, colorectal, esophageal, gastric, head and neck, kidney, liver, lung, lymphoma, pancreatic and prostate (**S1 Table** from Oncomine). Of the identified cancers, three (breast, colorectal and prostate) were selected for further analysis given the number of datasets found to have significant RARRES1 gene expression changes as well as the literature known about RARRES1 and its molecular biology within these cancers. Within the cancers selected, individual datasets were chosen given their large sample sizes and subtype analyses. Thus, the The Cancer Genome Atlas (TCGA) Breast Cancer, TCGA Colorectal Cancer, and Grasso Prostate datasets were selected. The Grasso Prostate dataset was also uniquely selected for its metastatic vs. primary analysis[23]. For the TCGA Breast Cancer dataset, subtype analysis was stratified into ER+ vs. ER- status; PR+ vs. PR- status and Triple Negative (PR-, HER2- & ER-) vs. non-Triple Negative [24]. Metastatic vs. primary site analysis was performed in the Grasso Prostate dataset. All colorectal cancer types (rectal mucinous adenocarcinoma, colonadenocarcinoma, rectal adenocarcinoma, colon mucinous adenocarcinoma and cecum adenocarcinoma) identified in the TCGA dataset were also analyzed in comparison to their respective normal samples (**S1 File**) [25]

Genes positively or negatively co-expressed with RARRES1 were further identified using Oncomine with respect to each dataset of tumor vs. normal. The sets of co-expressed genes with respect to each dataset were pipelined into DAVID to identify enriched pathways (adjusted P<0.05) found in the Reactome and Kyoto Encyclopedia of Genes and Genomes (KEGG) databases[26,27]. Significantly enriched pathways common across all datasets were then identified. In addition, genes important in fatty acid metabolism pathway were further assessed for co-expression with RARRES1 with respect to cancer subtypes (e.g. ER+ vs ER-) via correlation coefficients (**S1 File**). Selected genes included the following: *PPARG*, *PPARA*, *PPARGC1A*, *SREBF1*, *FASN*, *SCD1* and *CPT1A*.

#### Transcription factor prediction

Promoter and non-coding regions of RARRES1 gene were obtained from Ensembl and UCSC genome browser [28,29]. For each non-coding region, the sequences (from both sources) were loaded as the query sequence to search for potential binding sites. The query was pipelined into Alggen PROMO to predict transcription factors that can bind to the non-coding regions [30] (**S2 Table**). The prediction was carried out considering only human transcription factors and transcription factors with a maximum of 15% matrix dissimilarity rate were chosen. The Software PROMO v3.0.2, (which utilizes TRANSFAC v6.4) was used. We then utilized Harmonizome database and examined publicly available data on ChIP sequencing and ChIP-ChIP to identify whether the predicted transcription factors were found physically bound to the promoter region of RARRES1 gene in cells or organisms [31,32].

### *In vitro* data analysis

Results are shown as the mean ± SD. Statistical significance was calculated by using GraphPad Prism (La Jolla, California). Student's *t*-test and *P* < 0.05 was accepted as significant value (***, p < 0.001; **, p < 0.01; *, p < 0.05). At least three biological replicates were done to confirm the results.

## Results

### RARRES1 regulates lipogenesis and lipid droplet accumulation

**Fig 1:**
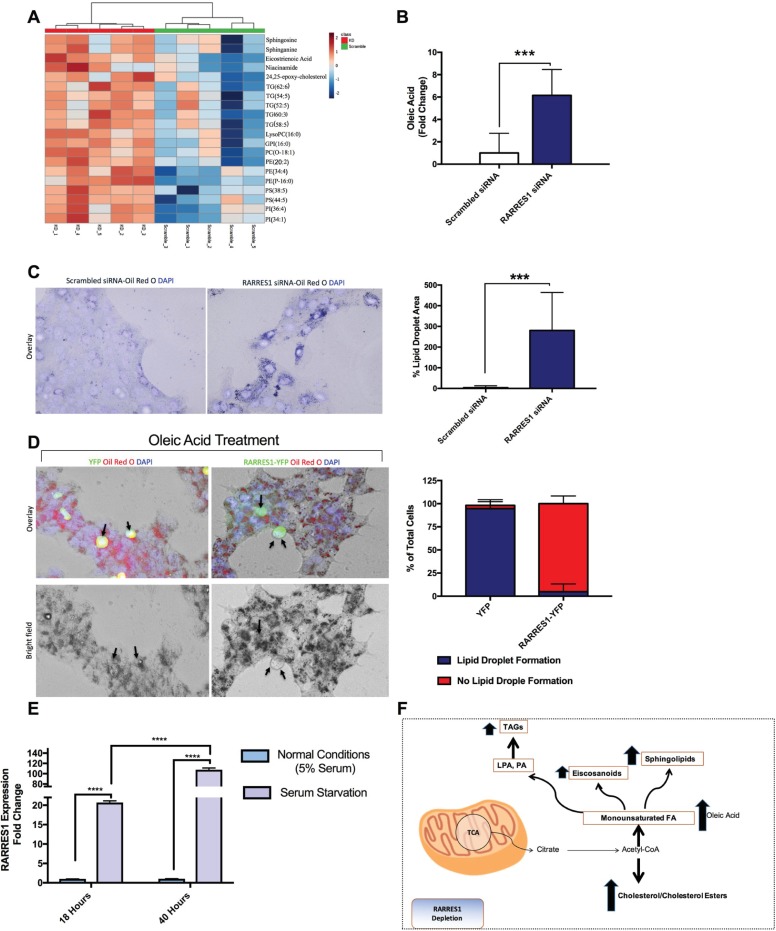
RARRES1 regulates lipid accumulation. **(A)** Classes of metabolites measured by non-targeted LC-MS analysis of transient RARRES1 knockdown MCF 10A cells and scrambled control cells. **(B)** GC-MS was run in transient RARRES1 knockdown MCF 10A cells and monounsaturated fatty acids were analyzed. Oleic acid is represented in the bar graph. **(C)** RARRES1 was transiently knocked down in MCF10A cells and Oil Red O staining was used to stain for lipid accumulation. The bar graph represents the intensity of lipid droplet staining. Three biological replicates are represented in the bar graph. **(D)** RARRES1-YFP was exogenously expressed and the transfected cells were treated with oleic acid and stained with Oil Red O to localize lipid droplets. The numbers of cells with YFP or RARRES1-YFP transfection displaying lipid droplets or no lipid droplets were counted in both HEK 293T cells and MCF 10A cells. The y-axis represents the % total of cells with lipid droplet formation (blue) and % cells with no lipid droplet formation (red). **(E)** MCF10A cells were serum starved for 18 hours or 40 hours. qPCR analysis was subsequently done. 18S was used as the endogenous control. **(F)** A schematic representation of metabolites that were upregulated in RARRES1-depleted cells.

Since RARRES1 expression is associated with metabolism-associated diseases and its exogenous expression regulates the expression of two important players in metabolic reprogramming (mTOR and SIRT1) [2], we examined whether RARRES1 has any functional significance in metabolic reprogramming. As RARRES1 is highly expressed in differentiated epithelial cells and is silenced in cancer cells [2,33–35], we used selected epithelial cells as our model. We subjected transient RARRES1-depleted mammary epithelial cells to non-targeted LC-MS (**Fig 1A and S1 Fig**) to get an overview of the metabolic changes that occur after decreasing the expression of the gene. We noticed a significant increase in neutral lipids, triacylglycerols and the cholesterol derivative 24,25-epoxy-cholesterol. Phospholipids, such as phosphatidylinositol (PI), phosphatidylethanolamine (PE), phosphatidylserines and phosphatidylcholines and the eicosanoid substrate, eicosatrienoic acid were also increased, as were three metabolites important in the synthesis of sphingolipids, sphinganine, niacinamide and sphingosine (**Fig 1A and S2 Fig**). These data indicate a global increase in lipid synthesis. We next used GC-MS to measure free monounsaturated and saturated fatty acids, since they are a good indicator of an increase in fatty acid synthesis [36]. We found an increase in oleic acid, the principle product of stearoyl CoA desaturase 1 (SCD1) (*SCD* gene), but no change in palmitate (**Fig 1B) (S3B and S3C Fig**) [37]. However palmitate, a saturated fatty acid, is highly toxic to the cell and is usually converted to palmitoleate or oleic acid [38]. We also examined lipid changes after RARRES1depletion in normal prostate epithelial cells and primary hepatocytes to ensure that this change in lipid content is not cell line specific, by GC-MS. In both cell lines there was increase in cholesterol and saturated fatty acids (stearic acid and myristic acid) (**S3D Fig**). Taken together these data demonstrate that manipulation of RARRES1 alters global fatty acid metabolism.

Since RARRES1 depletion increased several classes of lipids, including neutral lipids, we validated the findings by staining lipid droplets in cells. Scrambled siRNA transfected MCF 10A cells do not have visible lipid droplets but consistent with the LC/GC-MS data, transient RARRES1 depletion in normal breast epithelial MCF 10A cells, lead to significant accumulation of lipid droplets, verified by Oil Red O and Nile Red staining (**Fig 1C and S4A Fig**). We then transiently overexpressed RARRES1 tagged with YFP in MCF 10A and HEK-293T cells to visualize the transfected cells through fluorescence microscopy. We treated the cells with oleic acid for 4 hours or overnight (12 hours), respectively, to induce lipid droplets since these cells do not readily form visible lipid droplets in normal conditions. Cells that exogenously expressed RARRES1 had a striking decrease in lipid droplet accumulation compared to YFP expressing cells and the surrounding cells that did not take up the RARRES1-YFP plasmid (**Fig 1D and S4B and S4C Fig**). This indicates that higher levels of RARRES1 leads to the degradation or oxidation of exogenous fatty acids rather than their storage in lipid droplets. Conversely, depletion of RARRES1 leads to the accumulation of lipids. Consistent with this observation, RARRES1 expression in prostate and cervical cancer cells induces autophagy, a cellular response that sequesters and degrades lipid droplets during starvation, a mechanism called lipophagy [39].

Nutrient deprivation is also a major regulator of lipid metabolism [40]. If RARRES1 is an important regulator of lipid metabolism we considered if its expression (or activity) might change with nutrient deprivation. To test this, we starved MCF10A cells of serum for 18 and 40 hours and measured RARRES1 levels by qPCR. 24 hours after starvation, RARRES1 transcript levels were increased 10-fold over nutrient replete cells and over 100 fold at 48h hours (**Fig 1E**). We also assessed RARRES1 protein after 24-hour of serum starvation, and the results were consistent with the qPCR results (**S4D Fig**). It is important to note that the increase in RARRES-1 expression is only seen in normal epithelial cells. Cancer cells, in which *RARRES1* is silenced, cannot increase RARRES1 protein level after starvation. Taken together these data show that RARRES1 depletion in epithelial cells increases lipid content while RARRES1 overexpression decreases lipid accumulation, most likely through the upregulation of autophagy. We also show that RARRES1 expression is altered after serum starvation, an environment that triggers changes in lipid metabolism.

### RARRES1 depletion regulates *de novo* lipogenesis in epithelial cells

**Fig 2:**
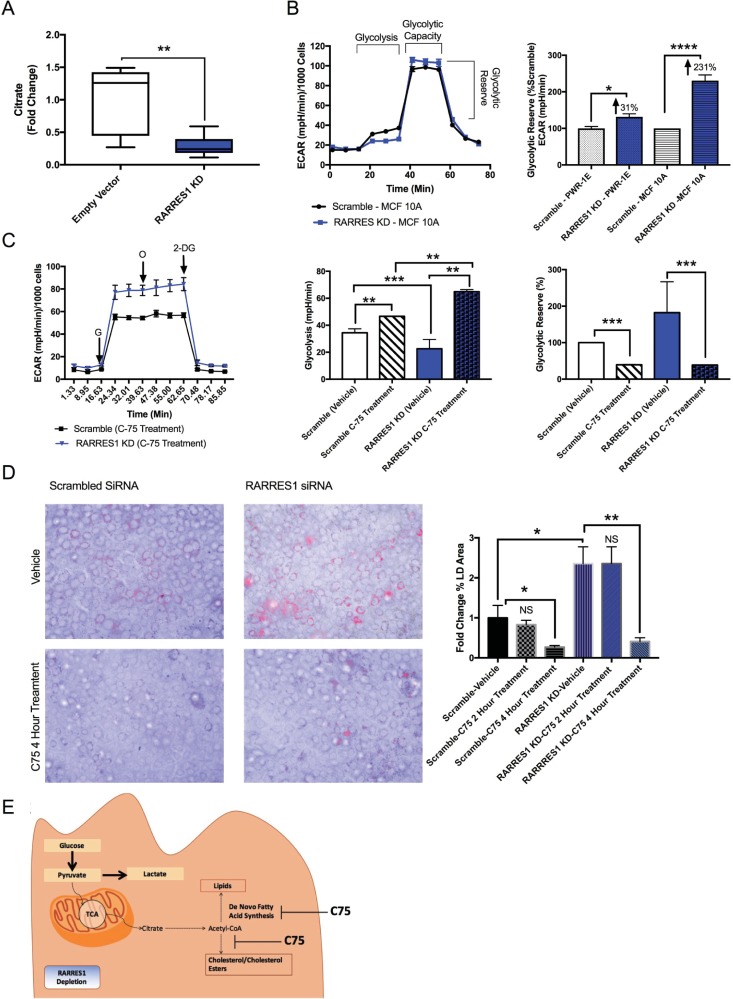
RARRES1 regulates DNL. **(A)** Citrate levels in stable RARRES1 knockdown MCF10A cells were calculated using MS/MS method and compared to empty vector MCF10A cells. **(B)** Glycolytic activity was assessed in MCF 10A and PWR-1E cells. The glycolytic reserve was calculated for both cell lines. **(C)** Transient RARRES1 KD and scramble control were treated with C75 inhibitor and glycolytic activity was assessed. Glycolytic usage and reserve were assessed in vehicle and C75 treated groups. **(D)** Scrambled siRNA or RARRES1 siRNA transfected cells were treated with vehicle or 40 μM C75 for 2 hours or 4 hours. Cells were then stained with Oil Red O and DAPI. **(E)** A schematic diagram of glycolysis and glucose dependent de novo lipogenesis pathway in RARES1 depleted epithelial cells and the effect of C75 on these cells is depicted. G: Glucose; O: Oligomycin; 2-DG: 2-Deoxy-D-glucose.

*De novo* fatty acid synthesis (lipogenesis) occurs when sugars, such as glucose, get converted to citrate and subsequently acetyl-CoA to produce cholesterol and the fatty acid metabolites that were enriched in RARRES1-depleted MCF 10A cells (**Fig 1A and 1B**) (**S3D Fig**) [41,42]. We thus assessed *de novo* lipogenesis (DNL) activity in RARRES1-depleted epithelial cells. Steady state citrate levels were decreased in stable RARRES1-depleted MCF 10A cells, thus indicating an increased rate of citrate conversion to acetyl-CoA or fatty acid metabolites (increased DNL) (**Fig 2A and S5 Fig**).

Since glucose is a major source for DNL, we assessed if RARRES1-depleted PWR-1E and MCF 10A cells undergo glycolytic reprograming to redirect glucose usage for *de novo* fatty acid synthesis [43]. Aerobic glycolysis was examined in RARRES1-depleted epithelial cells using the extracellular flux assay- Glycolysis Stress Test kit (Seahorse, Agilent Technologies, Santa Clara, CA, USA). Glycolytic usage was determined by measuring the extracellular acidification rate reached by a given cell after the addition of saturating amounts of glucose. Oligomycin was then injected to inhibit mitochondrial respiration and force the cell to use its glucose for glycolysis, which is observed by an increased extracellular acidification rate [44].This shift in ECAR is called the glycolytic capacity. Changes in glycolytic capacity were therefore assessed in RARRES1-depleted MCF 10A and PWR-1E cells. RARRES1 -depleted MCF 10A cells had a marked decrease in glucose usage but their capacity to induce glycolysis was higher than the control cells, which indicates that there is no glycolytic dysfunction (**S6A Fig**). The same glycolytic capacity phenomenon was also observed in PWR-1E cells (**S6B Fig**). We next measured the glycolytic reserve which is the difference between basal glycolysis and the glycolytic capacity. This determines the glucose that is not used in the basal state. The glycolytic reserve can provide insight on whether glucose is stored or shuttled to other pathways besides aerobic glycolysis. In both cell lines their glycolytic reserve increased, which shows that there is an increase in glucose being redirected to pathways other than glycolysis (**Fig 2B**). Glucose can also be utilized for oxidative phosphorylation (mitochondrial respiration). We thus used the extracellular flux assay to measure oxygen consumption rate, which determines the mitochondrial respiration activity. We first treated cells with glucose and after 12 minutes of incubation, cells were treated with oligomycin, the mitochondrial ATP synthase inhibitor, to measure glucose-mediated OCR (**S6C Fig**). There was no difference between scrambled siRNA and RARRES1 siRNA transfected cells. This indicates that RARRES1-depleted cells do not redirect their glucose for oxidative phosphorylation or aerobic glycolysis. Instead, glucose is being reprogrammed for other pathways.

The decrease in citrate levels, increase in lipid synthesis and glycolytic reprogramming in RARRES1- depleted epithelial cells suggest that glucose is mostly likely used for glucose- dependent DNL (**Figs 1 and 2A) (S6 Fig**). To test this, we used the fatty acid synthase inhibitor C75, which has been noted to markedly decrease lipid content by inhibiting fatty acid synthesis and increase citrate levels at a concentration of 40 μM [45,46]. We next examined whether C75 treatment can reverse the glycolytic reprogramming seen in RARRES1-depleted cells. We treated RARRES1-depleted MCF 10A cells with C75 for 1 hour or 2 hours at a concentration of 40 μM. In C75 treated MCF 10A cells, both control and RARRES1-depleted cells increased glycolytic usage and decreased their glycolytic reserve compared to the vehicle treated groups (**Fig 2C**). These observations indicate that MCF 10A cells have a significant basal level of DNL. Importantly, treatment of RARRES1-depleted cells with C75 completely reversed the decrease in glucose usage seen in the vehicle-treated cells and they had higher aerobic glycolytic activity (**Fig 2C**). C75 also reversed the changes in glycolytic reserve seen in the transient knockdown, as the glycolytic reserve is indistinguishable from the control group treated with 40 μM C75 (**Fig 2C**). We also assessed lipid droplet accumulation in RARRES1-depleted cells after treatment with 40 μM C75. C75 treatment reversed lipid droplet accumulation after 4 hours of treatment (**Fig 2D and S6D Fig**). This indicates that RARRES1-depleted cells use glucose for fatty acid synthesis instead of aerobic glycolysis and that inhibition of FAS by C75 reverses this phenotype (**Fig 2E**) and the accumulation of lipid droplets in RARRES1-depleted cells.

### RARRES1 depletion enhances substrate availability for fatty acid oxidation during starvation

**Fig 3:**
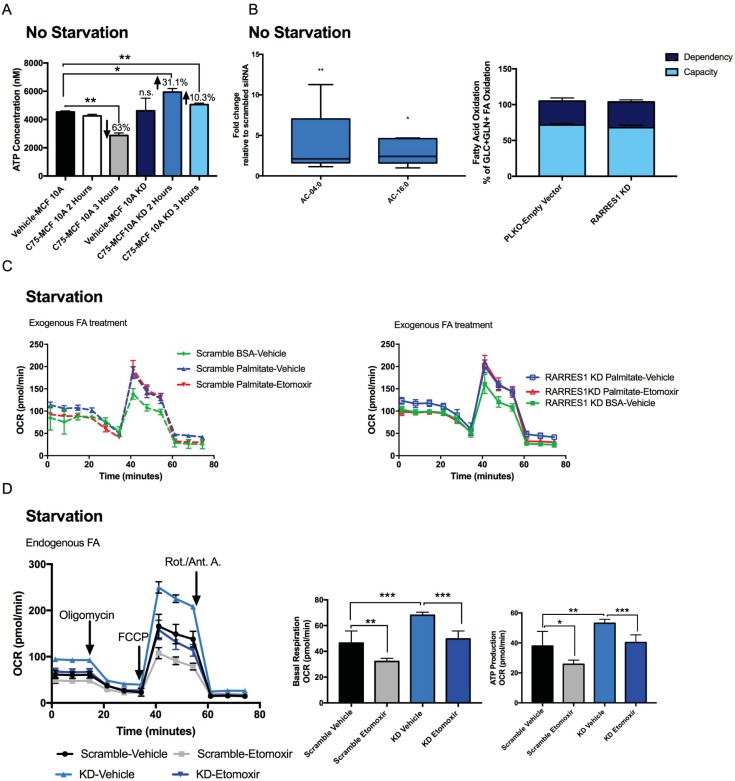
RARRES1 depletion regulates fatty acid oxidation. **(A)** ATP content was quantified in transient RARRES1 KD MCF 10A cells and scrambled siRNA MCF 10A cells after 2 and 3 hours of 40 μg/mL C75 treatment. **(B)** All detected Acylcarnitines, AC-4:0 and AC-16:0, were quantified and validated in the non-targeted LC-MS results of RARRES1 siRNA transfected MCF 10A cells **(S2A Fig)**. The fold change was normalized to scrambled siRNA transfected MCF 10A cells. Fatty acid oxidation rate in RARRES1-depleted cells was measured in nutrient rich media. FAO dependency and flexibility of the cells were calculated. Initial inhibition of FAO by etomoxir measures how dependent the cells are on that particular fuel source to meet the energy demand. Using a combination of glycolytic, glutaminolytic and FAO inhibitors, the cells' capacity and flexibility in meeting energy demand were calculated in terms of oxygen consumption rate. **(C)** Fatty acid oxidation dependent mitochondrial respiration was quantified and the effects of exogenous fatty acids (palmitate treatment) on FAO-dependent OCR in RARRES1-depleted and scrambled siRNA MCF 10A cells in glucose and serum starved media. **(D)** MCF 10A were starved and treated with etomoxir to measure fatty acid oxidation rate dependent on endogenous fatty acids. Scrambled siRNA and transient RARRES1 siRNA were measured and compared. Basal respiration and ATP production were quantified.

Several studies have shown that treatment with C75, at a concentration of 30 to 60 μg/mL, for 1 to 2 hours, decreases fatty acid oxidation (FAO), which is the incorporation of acetate into fat and increase in ATP production through palmitate oxidation [46–48]. We next examined whether C75 has any effect on ATP content of RARRES1-depleted cells compared to RARRES1-expressing cells. After 2 hours of treatment with 40 μg/mL C75, ATP content was significantly increased in RARRES1-depleted cells compared to C75-treated control cells (**Fig 3A**). The RARRES1-depleted cells were also capable of increasing ATP content after 3 hours of treatment unlike the control cells, in which a significant decrease in ATP concentration occurred at this time. This indicates that even though the RARRES1-depleted cells have more fatty acids available to oxidize for FAO, the cells in nutrient-rich conditions instead store the fatty acids until FAO is triggered by C75.

Next, FAO was directly measured to confirm that fatty acids were preferentially stored rather than oxidized in normal conditions as indicated in our ATP data (**Fig 3A**). Acylcarnitines are involved in the first committed step of FAO. Long chain fatty acids are catabolized to acetyl- coenzyme A, a major fuel for mitochondrial respiration (especially during starvation), acyl-CoAs must then be converted to acylcarnitine to cross the outer mitochondrial membrane [49]. Disorders of fatty acid metabolism are typically associated with primary and secondary forms of carnitine deficiency [50]. Thus monitoring carnitine levels is an accurate way to assess transportation of long chain fatty acids into mitochondria. Acylcarnitines were detected and identified in our non-targeted LC-MS analysis of MCF 10A cells in which RARRES1 was transiently depleted. Isobutyryl-L-carnitine (AC-4:0) and L-palmitoylcarnitine (AC-6:0) were increased in the transient knockdowns (**Fig 3B**). The data suggests that RARRES1 depletion increases the uptake of fatty acids into the mitochondria, either for beta oxidation or their diversion to the cytoplasm for fatty acid elongation. To address this, we examined fatty acid oxidation activity. FAO is a pathway that breaks down fatty acids into acetyl-CoA and subsequently feeds into the tricarboxylic acid (TCA) cycle for oxidative phosphorylation (mitochondrial respiration) [12]. The dependency on fatty acid oxidation was assessed by measuring OCR and treating cells with the fatty acid oxidation inhibitor (etomoxir) through the extracellular flux assay. There was no notable change in fatty acid oxidation dependency between control and RARRES1-depleted cells (**Fig 2B**). As suggested in Fig 2A, RARRES1 does not affect fatty acid oxidation activity in normal conditions. This suggests that during normal conditions (high glucose (10 mM) and serum (5%)), fatty acyl-carnitines are most likely utilized in the mitochondria for complex fatty acid synthesis.

As was the case with C75 treatment, during glucose and serum starvation, cells redirect their glucose dependent mitochondrial respiration to fatty acid oxidation. Because changes in ATP content were seen in RARRES1 depleted cells after C75 treatment, we monitored fatty acid dependent mitochondrial respiration in transient RARRES1 depleted MCF 10A and PWR-1E epithelial cells in starved conditions (0.5 mM glucose and 1% horse serum) (**Fig 3A**). MCF 10A and PWR-1E cells were used to ensure that the changes in fatty acid oxidation are due to epithelial specific effects rather than MCF10A specific changes. We first monitored the utilization of exogenous fatty acids for FAO by treating the cells with exogenous palmitate or BSA (as a control). This experiment ensures that RARRES1-depleted cells do not influence uptake of fatty acids for FAO. As expected, the OCR of RARRES1-depleted cells was indistinguishable from control cells after treatment with exogenous fatty acids (**Fig 3C**). We next examined the utilization of endogenous fatty acids for FAO during starvation. We depleted RARRES1 in PWR-1E and MCF 10A cells in normal conditions (10 mM glucose and 5% serum). 24 hour after transfection, fatty acid oxidation was triggered by depleting media of glucose and serum (1% serum and 0.5 mM glucose). RARRES1-depleted cells had a distinct oxygen consumption pattern compared to control cells. RARRES1 depleted MCF 10A cells had an increase in mitochondrial respiration that was reversed by treatment with the FAO inhibitor etomoxir (**Fig 3D**). The normal metabolic needs of prostate epithelial cells are different than mammary epithelial cells. For example, prostate tissue produces and secretes a significant amount of citrate instead of diverting it towards production of endogenous fatty acids through DNL [51]. In turn, they rely on exogenous fatty acids to induce fatty acid oxidation. Specifically during glucose starvation, they rely solely on exogenous fatty acids to produce ATP through mitochondrial respiration [52]. However, even in these cells, RARRES1 depletion significantly reversed the phenotype in glucose and serum starved conditions (**S7A Fig**).

These data demonstrate that cells in which RARRES1 expression is reduced, a phenomenon that occurs in (cancer cells, where the gene is silenced or suppressed, exposure to nutrient replete conditions (or treatment with palmitate) does not trigger fatty acid oxidation. Instead, RARRES1 depletion renders cancer cells more energetic when starved or treated with C75, mechanisms that trigger fatty acid oxidation, due to the increase in lipid substrates available for fatty acid oxidation. As transient starvation is a common phenomenon that occurs during nutrient deprivation and in the center of large tumors, our data suggest that cells may adapt to these harsh environments by reducing RARRES1 levels.

### RARRES1 gene expression in common solid tumors correlates with fatty acid metabolism genes

**Fig 4:**
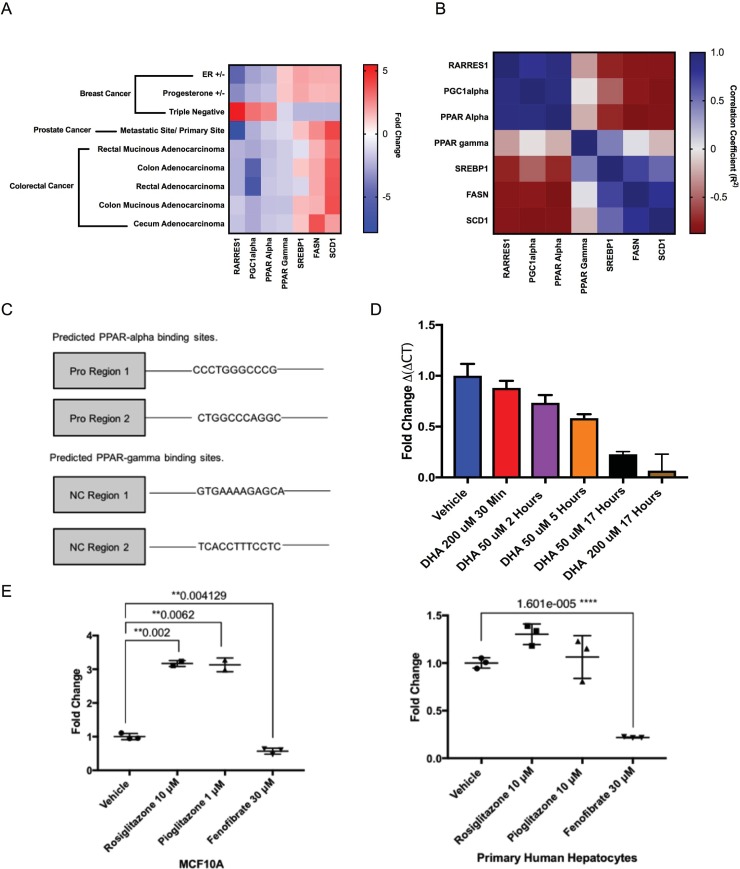
Differential expression of *RARRES1* in cancers correlates with expression of fatty acid metabolism genes. Cancers where *RARRES1* gene was differentially expressed were chosen and correlative analysis was done. *FASN*, *SCD*, *PPARG*, *SREBF1*, *PPARGC1A* and *PPARA*, were chosen as candidate genes important in fatty acid oxidation and lipogenesis. **(A)** Three subtypes of breast cancer, 5 types of colorectal cancer and metastatic versus primary sites of prostate cancer were analyzed and fold change difference (with p-value <0.01) was plotted for each gene. **(B)** The correlative score (calculated using Pearson correlation formula) was calculated between two genes in all cancers analyzed. **(C)** Alggen PROMO software predicted PPAR alpha and gamma in complex with RXR to bind to non-coding regions of *RARRES1* gene (**S2 Table**). **(D)** Endogenous PPAR agonist, DHA, regulates RARRES1 expression in MCF 10A cells. **(E)** Synthetic agonists of PPAR alpha (Fenofibrate) and gamma (Rosiglitazone and Pioglitazone) were used to treat MCF10A cells or primary hepatocytes for 48 hours.

In addition to its role in hepatosteatosis and other metabolic disorders, RARRES1 is among the most commonly methylated genes in multiple human tumors [53]. As our data indicate that RARRES1 has a role in fatty acid metabolism, we wondered if the extensive transcriptomic data available for multiple cancers also points to a relationship with fatty acid metabolism pathways. We used publicly available cancer datasets to assess whether *RARRES1* is co-expressed with fatty acid metabolism genes. Oncomine^TM^ (Compendia Bioscience, Ann Arbor, MI, USA) was used to identify cancers in which *RARRES1* is significantly increased or decreased (**S1 Table from Oncomine**). Of the identified cancers, three (breast, colorectal and prostate) were selected for further analysis given the number of datasets found to have significant RARRES1 gene expression changes. Interestingly, subtype analysis revealed differential RARRES1 expression for more aggressive tumor phenotypes. For example, hormone-negative breast cancers (e.g. TNBC vs. non-TNBC, ER+ vs. ER-, PR+ vs. PR-) and metastatic prostate cancer have differential RARRES1 gene expression (**Fig 4A**).

We then sought to determine important pathways associated with RARRES1 expression. Genes co-expressed with *RARRES1* were identified via Oncomine (Compendia Bioscience, Ann Arbor, MI) with respect to each cancer dataset (original data file available upon request). The sets of co-expressed genes were pipelined into DAVID [54,55] for pathway enrichment analysis (adjusted P<0.05). Enriched pathways common across all three cancer datasets were identified (**Table 1**). As expected, the retinol metabolism and protein digestion pathways were enriched across all datasets indicating a role of RARRES1 in retinoic acid biochemistry [1] and cellular autophagy [2,3]. Importantly, pathways involved in lipid metabolism, synthesis and signaling were identified (**Table 1**). These include PPAR signaling, steroid hormone biosynthesis and regulation of lipolysis/fat digestion.

**Table 1:** Common pathways identified in the analysis of genes co-expressed with RARRES1 in breast, prostate, and colorectal cancers. Pathways that were significantly enriched and shared among the three cancer datasets (TCGA breast, TCGA Colorectal and Grasso Prostate Cancers) of genes co-expressed with RARRES1 were listed in the table. The number of genes identified in the pathways out of the total genes identified in the pathway was listed. Statistical analysis was applied for all datasets and the P-value and Benjamini-Hochberg value determined for each cancer dataset are included in the table. DAVID platform was used to identify the pathways relevant in the cancer datasets.

Pathways Enriched in all Datasets	Number of Genes (Breast; Colorectal; Prostate)/ (Total Genes in Pathway)	P-Value (Breast; Colorectal; Prostate)	Benjamini (Breast; Colorectal; Prostate)
ECM-receptor interaction	(40; 28; 48)/ 87	9.32E-09; 0.0035; 1.10E-08	8.85E-07; 0.032; 5.38E-07
Retinol metabolism	(27; 29; 30)/65	0.000034; 4.35E-06; 6.09E-04	0.00064; 9.05E-05; 0.0043
Salivary secretion	(31; 28; 40)/86	0.000175; 0.0029; 4.80E-05	0.0022; 0.022; 4.85E-04
PPAR signaling pathway	(25; 21; 32)/67	4.88E-04; 0.017; 1.79E-04	0.004; 0.091; 0.00164
Steroid hormone biosynthesis	(24; 25; 24)/58	0.00011; 4.66E-05; 0.012	0.0016; 6.17E-04; 0.054
Regulation of lipolysis/ Fat Digestion	(24; 15; 24)/56	0.0000581; 0.0081; 0.0076	0.00092; 0.052; 0.38
PI3K-Akt signaling pathway	(98; 83; 124)/345	0.000003; 0.0085; 1.17E-05	0.000078; 0.054; 1.71E-04
Protein digestion and absorption	(40; 41; 41)/80	0.00000001381; 6.14E-09; 3.61E-05	0.00000363; 4.47E-07; 4.06E-04

We next sought to assess the correlation of *RARRES1* with important genes implicated in PPAR signaling and fatty acid metabolism. These genes include *PPARG*, *PPARGC1A*, *PPARA*, *SCD*, *FASN* and *SREBF1*. *SREBF1* or Sterol regulatory element-binding transcription factor 1 (SREBP-1) protein is a transcription factor activated by mTOR which subsequently upregulates genes including *SCD or* stearoyl-CoA desaturase 1 (SCD1) enzyme and *FASN* or fatty acid synthase (FAS) that are essential in lipogenesis [56]. The interaction of peroxisome proliferator-activated receptor alpha (PPARα) protein or *PPARA* gene with peroxisome proliferator-activated receptor gamma coactivator 1-alpha (PGC1α) protein or *PPARGC1A* gene induces the transcription of genes that are important in fatty acid oxidation [57]. These genes correlate with RARRES1 gene expression changes when all cancers are considered (**Fig 4A**). For example, *PPARGC1A* and *PPARA* positively correlated with RARRES1 whereas *FASN* and *SCD* negatively correlated with *RARRES1* gene expression. This correlation pattern was further recapitulated in all individual subtype analyses (e.g. TNBC vs. non-TNBC) (**Fig 4B**). *RARRES1* expression therefore is positively correlated with fatty acid oxidation genes (*PPARGC1A*, *PPARA*) and negatively correlated with lipogenesis genes (*FASN*, *SCD*). Taken together these data demonstrate the association of RARRES1 and fatty acid metabolism in the context of cancer.

The peroxisome proliferating activated receptors correlated with RARRES1 expression are important in lipid accumulation, storage, transportation and oxidation of fatty acids. As PPARs are important transcriptional regulators (both negative and positive) of metabolism genes we wondered if they might be important RARRES1 regulators. ALGEN PROMO was used to predict whether PPAR transcription factors can bind on the non-coding regions of RARRES1 gene [30]. This includes the promoter region and the introns of the gene. PPAR gamma or PPAR alpha form heterodimers with retinoid x receptor (RXR) and regulate transcription of genes involved in insulin action, adipocyte differentiation, lipid metabolism and inflammation [58,59]. Peroxisome proliferating activated receptor alpha and gamma (PPARα and PPARγ) in complex with RXR were predicted to bind to the non-coding regions of RARRES1 gene (**Fig 4C & S2 Table).** In addition, examination of the publicly available ChIP-X database, a web-based curated and interactive application that includes ChIP-chip, ChIP-seq, ChIP-PET and DamID studies in order to identify genome binding sites and target genes of transcription factors, for RARRES1 binding transcription factors clearly shows that PPARs are found in association with the RARRES1 promoter [31,32,60,61].

We thus evaluated whether PPAR signaling and treatment with their fatty acid ligands can regulate the expression of RARRES1. As noted earlier, RARRES1 is silenced in cancer cells; therefore normal epithelial cells, where RARRES1 is endogenously expressed, are the best models to use to assess whether PPARs can regulate the expression of RARRES1. We first treated in MCF 10A and PWR-1E cells with an omega-3 fatty acid, docosahexaenoic acid (DHA), an endogenous agonist of PPAR α and PPAR γ [62]. There was a rapid decrease in RARRES1 transcription after 30 minutes of treatment and a further decrease in its transcription at later times in MCF 10A cells (**Fig 4D**). RARRES1 expression in PWR-1E cells also responded to DHA (**S7B Fig**). We then treated cells with synthetic PPARγ and PPARα agonists to identify, which PPAR isotype regulates RARRES1 expression. We observed induction of RARRES1 transcription by PPARγ agonists, rosiglitazone and pioglitazone, and reduction in RARRES1 expression with fenofibrate, a PPARα agonist, after 48 hours of treatment (**Fig 4E**). Primary hepatocytes did not respond to rosiglitazone and pioglitazone because they do not express PPARγ (**Fig 4E**). This also suggests that PPARγ agonists regulate expression through PPARγ and not by a non-specific effect. Rosiglitazone and pioglitazone are known to induce lipid droplets whereas fenofibrate increases fatty acid oxidation and decreases lipid droplet content [63]. RARRES1 could be regulated to attenuate the metabolic effects that are induced by these agonists. Taken together, we show that RARRES1 is contextually correlated with fatty acid metabolism genes in common solid tumors and we subsequently show that two of these RARRES1 correlated genes (PPARs) modulate the expression of RARRES1.

## Discussion

**Fig 5:**
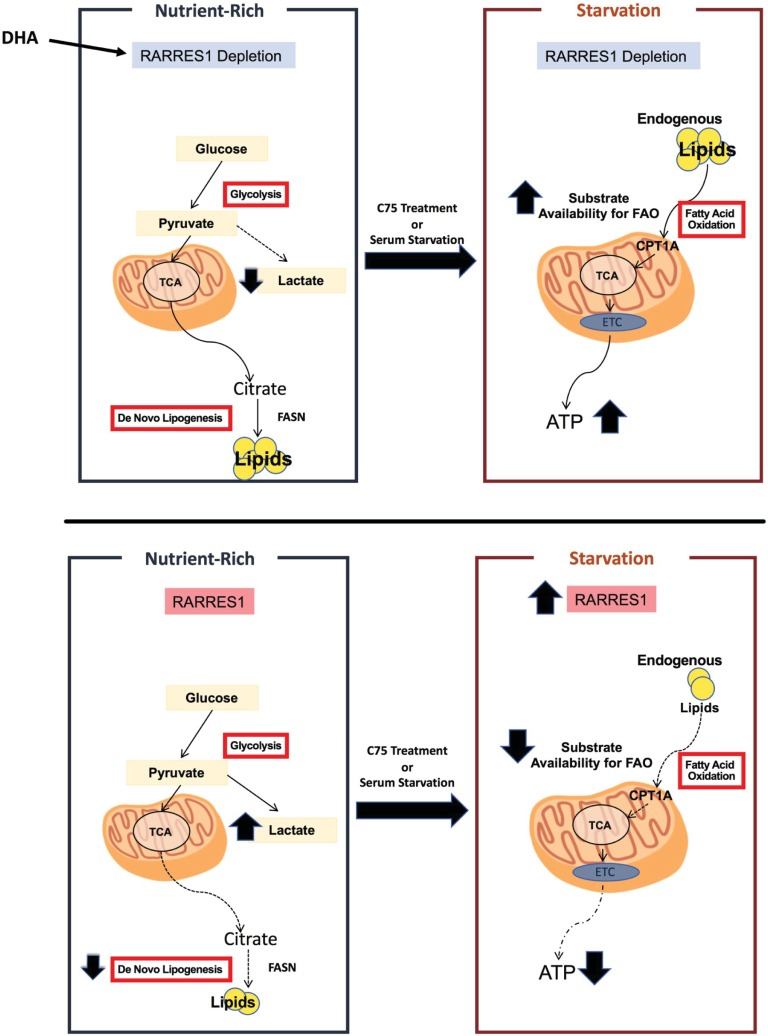
Effects of RARRES1 depleted vs. RARRES1-expressing epithelial cells on lipid metabolism. Unlike RARRES1 expressing cells, RARRES1 depleted cells increase *de novo* lipogenesis. This effect enables cells to improve mitochondrial respiration during starvation due to an increase in endogenous fatty acid availability. In RARRES1 expressing epithelial cells, glucose is mostly directed to lactate production in normal conditions. During serum starvation expression of RARRES1 increases and exasperates the lipid availability for fatty acid oxidation. ETC: Electron transport chain; TCA: tricarboxylic acid cycle; DHA: docosahexaenoic Acid.

In this study, we have shown that RARRES1 depletion in normal epithelial cells increases lipid content while RARRES1 overexpression decreases lipid accumulation. Second, we demonstrate that the increase seen in RARRES1-depleted cells is through glucose-dependent *de novo* lipogenesis. The phenotype is reversed when RARRES1-depleted cells are treated with C75, a lipogenesis inhibitor. Third, we find that RARRES1-depleted cells do not trigger fatty acid oxidation in nutrient replete or palmitate supplemented media. Instead, RARRES1 depletion primes the cells to have a more energetic phenotype during starvation due to the increase in endogenous fatty acid substrates available for FAO (**Fig 5**). This phenotype is reversed when cells are treated with the FAO inhibitor, etomoxir. RARRES1 expression was also contextually correlated to fatty acid metabolism genes in multiple cancer types. We demonstrated that two of these genes, peroxisome proliferating activated receptor alpha and gamma (PPARα and PPARγ) regulate RARRES1 expression in epithelial cells. These findings identify RARRES1 as a novel regulator of lipid metabolism.

Our data is supported by examination of publicly available databases that have listed RARRES1 as one of many genes regulated in metabolic diseases. For example RARRES1 is the most up-regulated gene in subcutaneous fat from obese human subjects on a diet-induced weight loss regimen, and is among the most downregulated genes in adipose tissue during weight maintenance in the obese human subjects [4]. Examination of deposited microarray data from a study focusing on hyperinsulinemia indicated that RARRES1 was also markedly decreased after insulin treatment in human skeletal muscles [64]. RARRES1 increased during dedifferentiation of adipocytes and decreased during differentiation and is differentially expressed in mouse models of hepatic steatosis and cholestatic liver disease [6,7,65]. Our study is the first to demonstrate a direct role for RARRES1 in the changes of lipid metabolism associated with these metabolic diseases.

RARRES1 is silenced in colorectal cancer, prostate cancer, nasopharyngeal cancer, Wilms tumor and leukemia [8,35]. Colorectal cancer and prostate cancer cell lines transfected with RARRES1 were less invasive and more apoptotic [66]. These findings support the idea of RARRES1 as a tumor suppressor, although this has not been formally demonstrated in animals. However, RARRES1 is increased in some mesenchymal-like cancers such as triple negative breast cancer [10,53] (**Fig 4A**). The present study demonstrates an important role for RARRES1 in fatty acid metabolism and may explain the duality of RARRES1 in cancer etiology. The role of fatty metabolism on cancer progression is also contextual. During nutrient deprivation, fatty acid degradation is necessary and fatty acid synthesis, specifically DNL, is increased in metastasis and certain cancers like prostate cancer, consistent with the decrease of RARRES1 expression in prostate cancer [51,67,68]. There is also evidence of changes in fatty acid metabolism preference in different subtypes of breast cancer, in which RARRES1 is differentially expressed. For example, fatty acid oxidation is disproportionately dysregulated in triple negative breast cancers compared to other subtypes of breast cancer [69]. Hormone receptor status in breast cancer also correlates with changes in lipid metabolism [70].

In summary, this study demonstrates that RARRES1 is a novel regulator of fatty acid metabolism in epithelial cells and points to an important role in diseases in which lipid metabolism is a hallmark of disease progression. Treatments that regulate RARRES1 expression or activity could have utility in obesity, cholestatic liver disease, heart disease (in which RARRES1 expression is markedly decreased in hypertrophic and dilated cardiomyopathy) and cancers where lipogenesis is crucial for the progression of certain tumors [71].

## Supporting information

S1 Fig Non-targeted LC-MS analysis of transient RARRES1 KD in MCF 10A cells. **(A)** Heat map of all metabolites significantly altered were depicted. **(B)** Important features in transient RARRES1 KD MCF 10A cells were selected by volcano plot with fold change threshold (x) 2 and t-tests threshold (y) 0.05. The red circles represent features above the threshold. Not the fold changes are log transformed. The further its position away from (0,0), the more significant the feature is.
(TIFF)

S2 Fig Metabolites detected in positive and negative mode LC-MS that were significantly altered in transient RARRES1 knockdown were validated through peak matching. Above each graph, the lipid classification (name), mass/charge (m/z), retention time (RT), and type of adduct (M±ion), depicted as name; m/z_RT; M±ion; are included. Only metabolites with significant changes, in terms of scramble vs. RARRES1 knockdown, were validated. **(A)** The identity of the metabolite was validated using tandem mass spectrometry. The daughter and parent ions for the metabolites were matched with the MS/MS spectra available in HMDB, SimLipid software V6.01 (Premier Biosoft, Palo Alto, CA, USA) and LIPID MAPS [17–19]. **(B)** Additional validations for lipids were done through SimLipid software using MSE data.
(PDF)

S3 Fig GC-MS analysis. **(A)** Important features in transient RARRES1 KD MCF 10A cells were selected by volcano plot with fold change threshold (x) 2 and t-tests threshold (y) 0.05. The red circles represent features above the threshold. Not the fold changes are log transformed. The further its position is away from (0,0), the more significant the feature is. **(B)** Oleic acid GC-MS fragmentation pattern peaks in transient RARRES1 KD and scramble MCF 10A cells were aligned against the fragmentation pattern peaks available in NIST database. **(C)** Detected palmitic acid was quantified and normalized against the peak intensity of the scramble control. **(D)** Stearic acid, myristic acid and cholesterol were detected in GC-MS and quantified in transient RARRES1 KD PWR-1E and primary human hepatocytes. The fold change is in terms of the peak intensity of the corresponding metabolites in the appropriate scrambled siRNA transfected control cells.
(TIFF)

S4 Fig RARRES1 regulates lipid content. **(A)** Transient RARRES1 KD MCF 10A cells were stained with Nile Red to validate the Oil Red O staining results in **Fig 1C**. Oleic acid treatment was used as a positive control. **(B)** RARRES1-YFP was overexpressed or YFP (negative control) in oleic acid treated MCF 10A cells and droplets were stained with Oil Red O. **(C)** DAPI staining of RARRES1-YFP and YFP overexpression in HEK 293T cells **(Fig 2B).** Arrows point at RARRES1-transfected or YFP-transfected cells in Fig 2B. **(D)** MCF 10A cells were either grown in nutrient rich media (labeled as control) or starved for 24 hours. Starved cells were also transfected with RARRES1 siRNA to ensure RARRES1 KD is efficient to perform experiments when cells are starved. Western blot was run to analyze the expression of RARRES1. Alpha-tubulin was used as the loading control. The band intensities of each sample was quantified using ImageJ and normalized to alpha-tubulin. The final fold change was based upon RARRES1 expression in control cells. Refer to **S9 Fig** for full-length blots.
(TIFF)

S5 Fig LC-MS of stable RARRES1 knockdown MCF 10A cells. Citrate was confirmed by comparing the retention time under the same chromatographic conditions and by matching the fragmentation pattern of the parent ion from the biological sample to that of the standard metabolite using tandem mass spectrometry (UPLC-TOFMS/MS). Citrate (or citric acid) peaks are displayed below the peaks of the predicted citrate metabolite in the cell extract.
(TIFF)

S6 Fig Glycolytic activity in RARRES1-depleted epithelial cells. **(A)** Glycolytic usage and capacity was quantified in transient RARRES1 knockdown in MCF 10A cells by using the Glycolysis Stress Test. **(B)** Oxygen consumption rate measurement was assessed after glucose injection in the Seahorse XF Flux machine. **(C)** Transient glycolytic activity was assessed in PWR-1E cells with transient RARRES1 knockdown by using the Seahorse Glycolysis Stress Test. Glycolytic usage and capacity was quantified.**(D)** RARRES1-siRNA or scrambled siRNA transfected cells were treated with vehicle (EtOH), or 40 μM C75 for 2 hours or 4 hours. Cells were stained with Oil Red O and DAPI.
(TIFF)

S7 Fig Fatty acid oxidation activity in RARRES1-depleted epithelial cells. **(A)** PWR-1E cells were starved (1% serum and 0.5 mM glucose) and treated with etomoxir to measure fatty acid oxidation rate dependent on endogenous fatty acids. Scrambled siRNA and transient RARRES1 siRNA were measured and compared. Basal respiration and ATP production were quantified. **(B)** PWR-1E cells were treated with DHA for 5 hours and 17 hours. qPCR was run to assess RARRES1 expression. 18S gene was used as the endogenous control.
(TIFF)

S8 Fig RARRES1-YFP and RARRES1 siRNA transfection efficiency. **(a)** RARRES1-YFP (expected band ~ 60 kDa) expression was validated in HEK 293 T cells. Tubulin or GAPDH was used as a loading control. The image was cropped, and lanes were juxtaposed; black line is drawn to describe the boundary. The full-length blot is presented in **S9A Fig.** RARRES1-YFP overexpression was also confirmed in MCF 10A cells. The full-length blot is presented in **S9B Fig**. **(b)** Western blot was done to confirm the transient RARRES1 knockdown efficiency in MCF 10A cells. The full-length blot is presented in **S9E Fig**. **(C)** qPCR was run to validate RARRES1 knockdown in PWR-1E cells. Transcript levels were normalized to the level of 18S. RARRES1 protein expression was confirmed in our previous study[33]. **(D)** Stable RARRES1 knockdown in MCF 10A cells were validated through RT-PCR. Beta-actin was used as a loading control. Full-length gel is presented in **S9C Fig**. **(E)** Stable RARRES1 knockdown was also confirmed through western blot. Alpha-tubulin was used as a loading control. Full-length blots are presented in **S9D Fig**.
(PDF)

S9 Fig Full-length gels. **(A)** RARRES1-YFP overexpression confirmation. We included transfection of truncated RARRES1-YFP and RARRES1 overexpression with no tag in order to assess the efficiency of transfection and validate that the full-length RARRES1 is being expressed. **(B)** Full-length blot of RARES1-YFP overexpression in MCF 10A cells is represented. NA (not applicable) was labeled on lanes that were irrelevant to the study. **(C)** RARRES1 knockdown efficiency was assessed. The full-length film with four different blots (examining different proteins (not relevant to this study)) is included. And the relevant blot was highlighted with black boundaries. Control cells with no transfection and scrambled siRNA and RARRES1 siRNA transfected MCF 10A cells were assessed. GAPDH was selected as a loading control. **(D)** Full-length blots of RARRES1 stable knockdown western blots are pictured. The left blot is probed for RARRES1 while the blot on the right is probed for alpha tubulin. The lanes that are relevant to this study are highlighted in black borders. **(E)** Full length gel of the stable RARRES1 knockdown MCF 10A cells. Empty vector was also transfected with RARRES1 siRNA to ensure the band is RARRES1-specific. Beta-actin was used as a loading control. Note: The third sample had to be loaded to a new lane (4^th^ band) due to technical issues that occurred while loading sample the first time (3^rd^ band). (**F)** Full length blot of the effects of serum starvation on RARRES1 western blots are pictured. The top blot is probed for RARRES1 while the blot on the bottom is probed for alpha tubulin.
(PDF)

S1 Table Oncomine Analysis: Cancer Summary for RARRES1 Expression. The Oncomine database was queried for RARRES1 expression in the available datasets based on the following criteria: cancer type, cancer versus normal, cancer versus cancer, cancer subtype, cancer versus baseline, pathway and drug and outlier analyses. The 'red' cells represent RARRES1 overexpression and the 'blue’ cells represent RARRES1 underexpression. The levels of expression are based on the gene rank percentile. This disease summary was performed using a criterion of a 2-fold change for RARRES1 expression and a p-value of 1E-4. (Oncomine platform (C).
(TIFF)

S2 Table Prediction of PPAR-gamma:RXR-alpha and PPAR-alpha:RXR-alpha binding to Non-coding Regions of *RARRES1* gene using Algen PROMO. In silico identification of PPAR-alpha and PPAR-gamma binding sites and within human *RARRES1* gene promoter and intron 1 (non-coding regions). Raw data are shown for PPAR-alpha and PPAR-gamma binding sites in RARRES1 non-coding regions. Numbering is based on the transcription start site at position 1000. PROMO displays RARRES1 and PPAR-gamma and -alpha as indicated, along with calculated start and end positions of transcription factor (TF) binding sites. Dissimilarity values give the percent difference in sequence similarity between the RARRES1 sequence and the calculated transcription factor (TF) consensus matrix. RE, or random expectation, yields the probability that the TF consensus binding sequence would happen by chance, where 0.1 signifies 1 incidence in every 10^4^ bases. Sequences for potential binding sites predicted by the software are included in the table.
(TIFF)

S1 File Expression Level of *RARRES1* and fatty acid metabolism genes in common solid tumors. Genes important in fatty acid metabolism pathway were further assessed for co-expression with RARRES1 with respect to cancer subtypes (e.g. ER+ vs ER-) via correlation coefficients. Within the cancers selected, individual datasets were chosen given their large sample sizes and subtype analyses. Thus, the The Cancer Genome Atlas (TCGA) Breast Cancer, TCGA Colorectal Cancer, and Grasso Prostate datasets were selected. The Grasso Prostate dataset was also uniquely selected for its metastatic vs. primary analysis [23]. For the TCGA Breast Cancer dataset, subtype analysis was stratified into ER+ vs. ER- status; PR+ vs. PR- status and Triple Negative (PR-, HER2- & ER-) vs. non-Triple Negative [24]. Metastatic vs. primary site analysis was performed in the Grasso Prostate dataset. All colorectal cancer types (rectal mucinous adenocarcinoma, colonadenocarcinoma, rectal adenocarcinoma, colon mucinous adenocarcinoma and cecum adenocarcinoma) identified in the TCGA dataset were also analyzed in comparison to their respective normal samples. Selected genes included the following: *PPARG*, *PPARA*, *PPARGC1A*, *SREBF1*, *FASN*, *SCD1* and *CPT1A*. Gene rank, p-value, t-test, q-test and fold change are included in the analysis.
(XLSX)

## Abbreviations

RARRES1: Retinoic acid receptor responder 1
FAO: fatty acid oxidation
DNL: *de novo* lipogenesis
mTOR: mechanistic target of rapamycin
SIRT1: sirtuin 1
*PPAR*G: peroxisome proliferator-activated receptor gamma
PPARA: peroxisome proliferator-activated receptor alpha
PPARGC1A: peroxisome proliferator-activated receptor gamma coactivator 1 alpha
SREBF1: sterol regulatory element-binding transcription factor 1
FASN: fatty acid synthase
SCD: Stearoyl-CoA desaturase
ETC: Electron transport chain
TCA: tricarboxylic acid cycle
