# Supplementary figures and images for: Tumor suppressor RARRES1- A novel regulator of fatty acid metabolism in epithelial cells
Source: PLoS One. 2018 Dec 17;13(12):e0208756. doi: 10.1371/journal.pone.0208756 (PMC6296515; doi:10.1371/journal.pone.0208756)

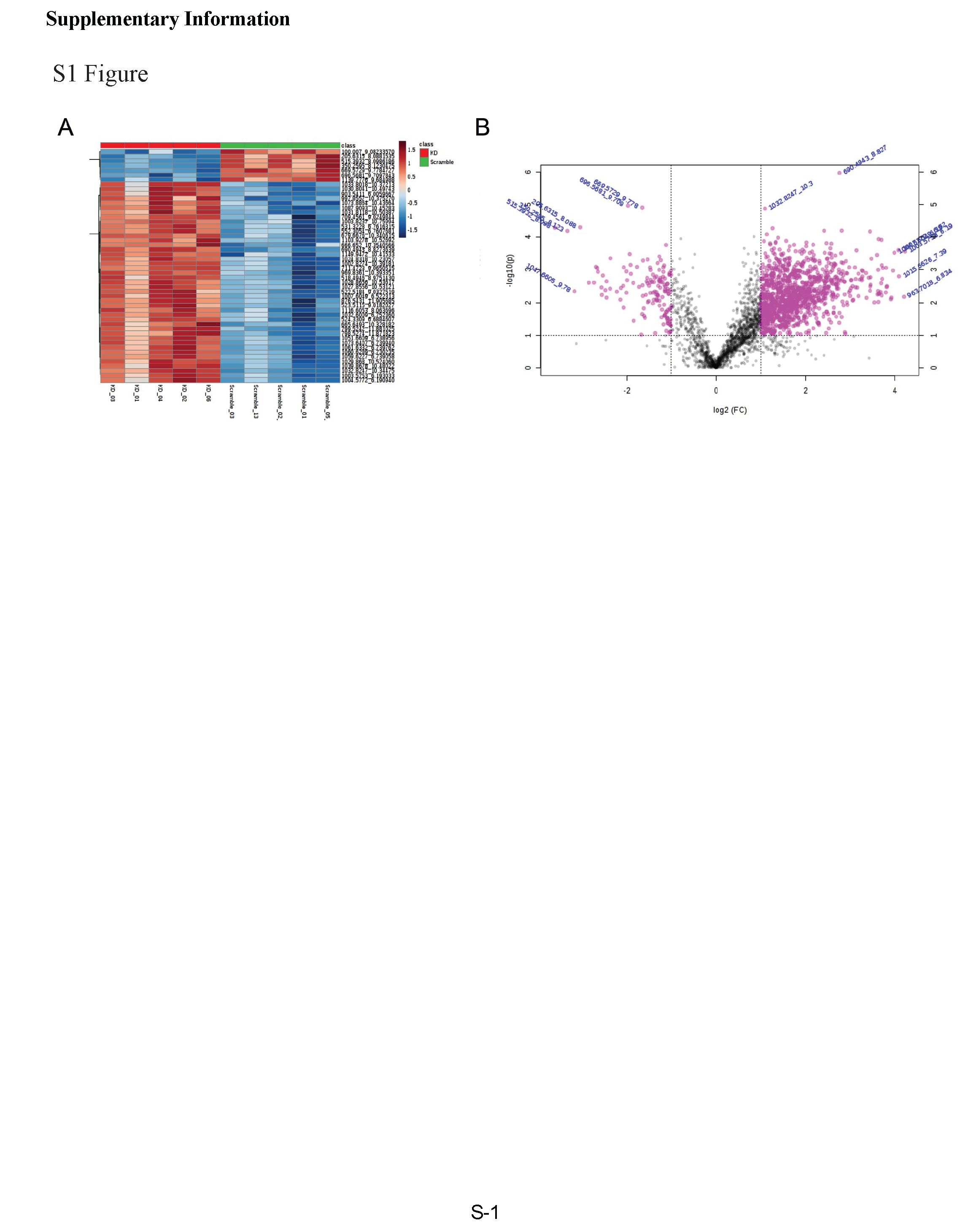

Supplement: S1 Fig — (A) Heat map of all metabolites significantly altered were depicted. (B) Important features in transient RARRES1 KD MCF 10A cells were selected by volcano plot with fold change threshold (x) 2 and t-tests threshold (y) 0.05. The red circles represent features above the threshold. Not the fold changes are log transformed. The further its position away from (0,0), the more significant the feature is. (TIFF) [file pone.0208756.s001.tiff]

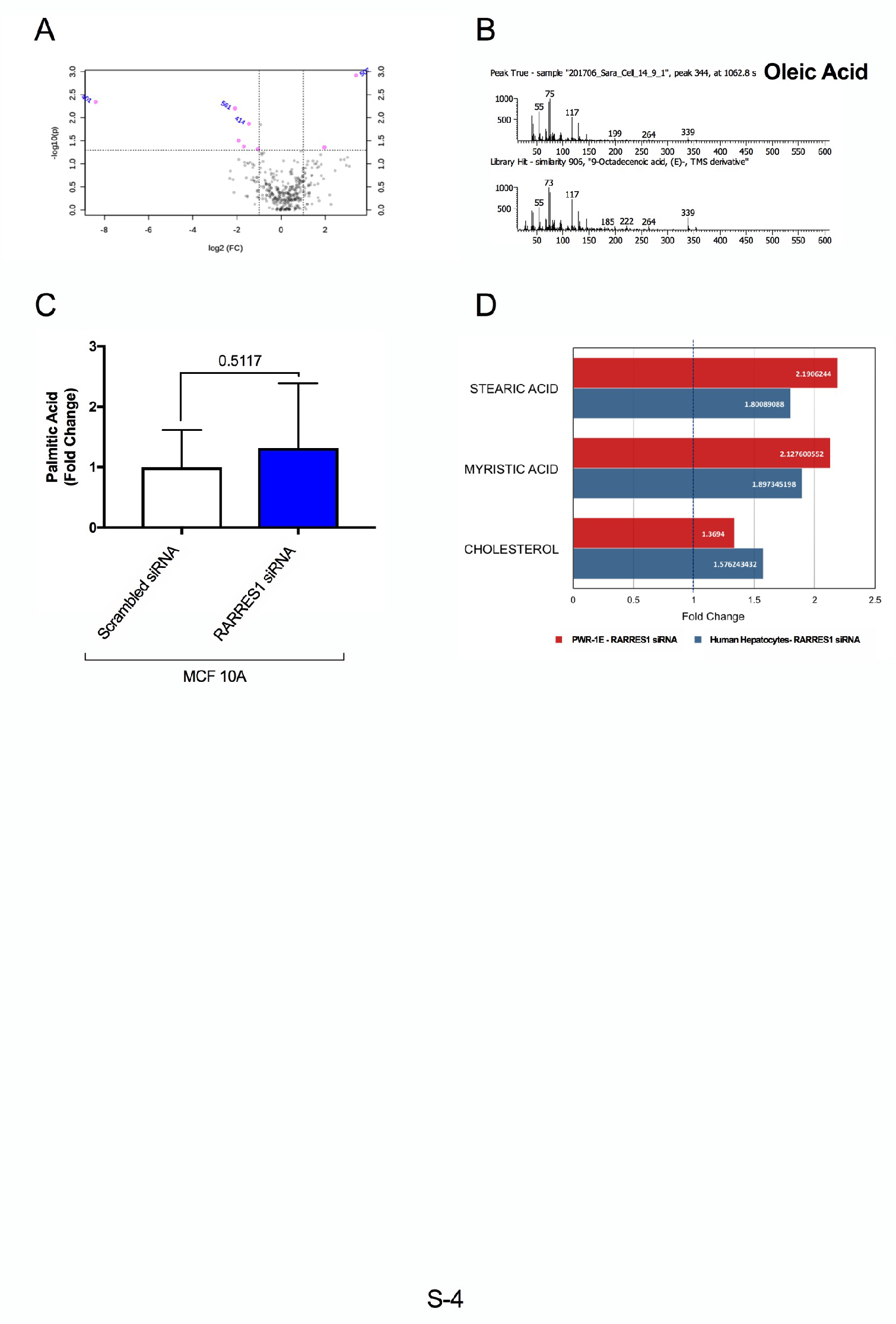

Supplement: S3 Fig — (A) Important features in transient RARRES1 KD MCF 10A cells were selected by volcano plot with fold change threshold (x) 2 and t-tests threshold (y) 0.05. The red circles represent features above the threshold. Not the fold changes are log transformed. The further its position is away from (0,0), the more significant the feature is. (B) Oleic acid GC-MS fragmentation pattern peaks in transient RARRES1 KD and scramble MCF 10A cells were aligned against the fragmentation pattern peaks available in NIST database. (C) Detected palmitic acid was quantified and normalized against the peak intensity of the scramble control. (D) Stearic acid, myristic acid and cholesterol were detected in GC-MS and quantified in transient RARRES1 KD PWR-1E and primary human hepatocytes. The fold change is in terms of the peak intensity of the corresponding metabolites in the appropriate scrambled siRNA transfected control cells. (TIFF) [file pone.0208756.s003.tiff]

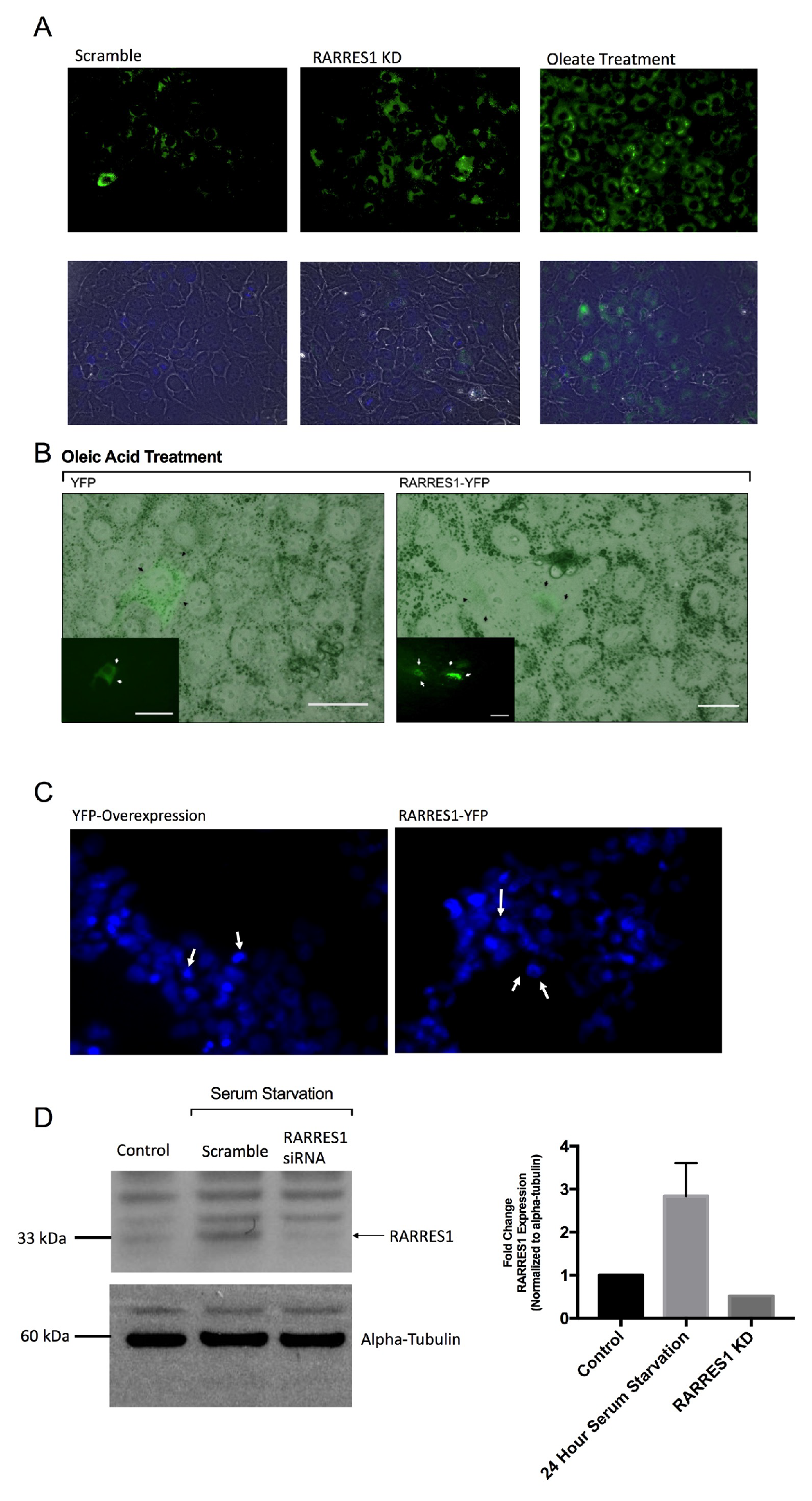

Supplement: S4 Fig — (A) Transient RARRES1 KD MCF 10A cells were stained with Nile Red to validate the Oil Red O staining results in Fig 1C. Oleic acid treatment was used as a positive control. (B) RARRES1-YFP was overexpressed or YFP (negative control) in oleic acid treated MCF 10A cells and droplets were stained with Oil Red O. (C) DAPI staining of RARRES1-YFP and YFP overexpression in HEK 293T cells (Fig 2B). Arrows point at RARRES1-transfected or YFP-transfected cells in Fig 2B. (D) MCF 10A cells were either grown in nutrient rich media (labeled as control) or starved for 24 hours. Starved cells were also transfected with RARRES1 siRNA to ensure RARRES1 KD is efficient to perform experiments when cells are starved. Western blot was run to analyze the expression of RARRES1. Alpha-tubulin was used as the loading control. The band intensities of each sample was quantified using ImageJ and normalized to alpha-tubulin. The final fold change was based upon RARRES1 expression in control cells. Refer to S9 Fig for full-length blots. (TIFF) [file pone.0208756.s004.tiff]

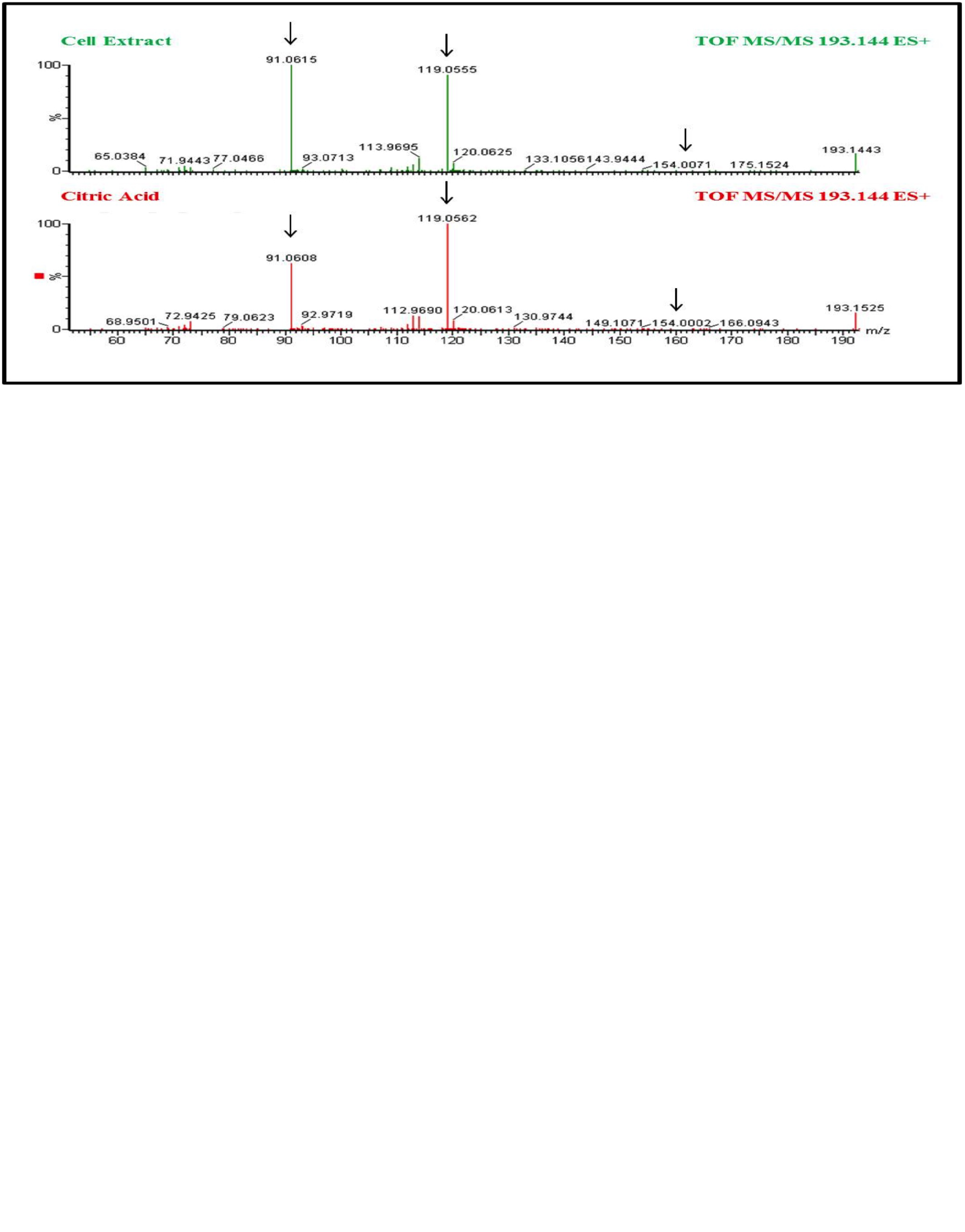

Supplement: S5 Fig — Citrate was confirmed by comparing the retention time under the same chromatographic conditions and by matching the fragmentation pattern of the parent ion from the biological sample to that of the standard metabolite using tandem mass spectrometry (UPLC-TOFMS/MS). Citrate (or citric acid) peaks are displayed below the peaks of the predicted citrate metabolite in the cell extract. (TIFF) [file pone.0208756.s005.tiff]

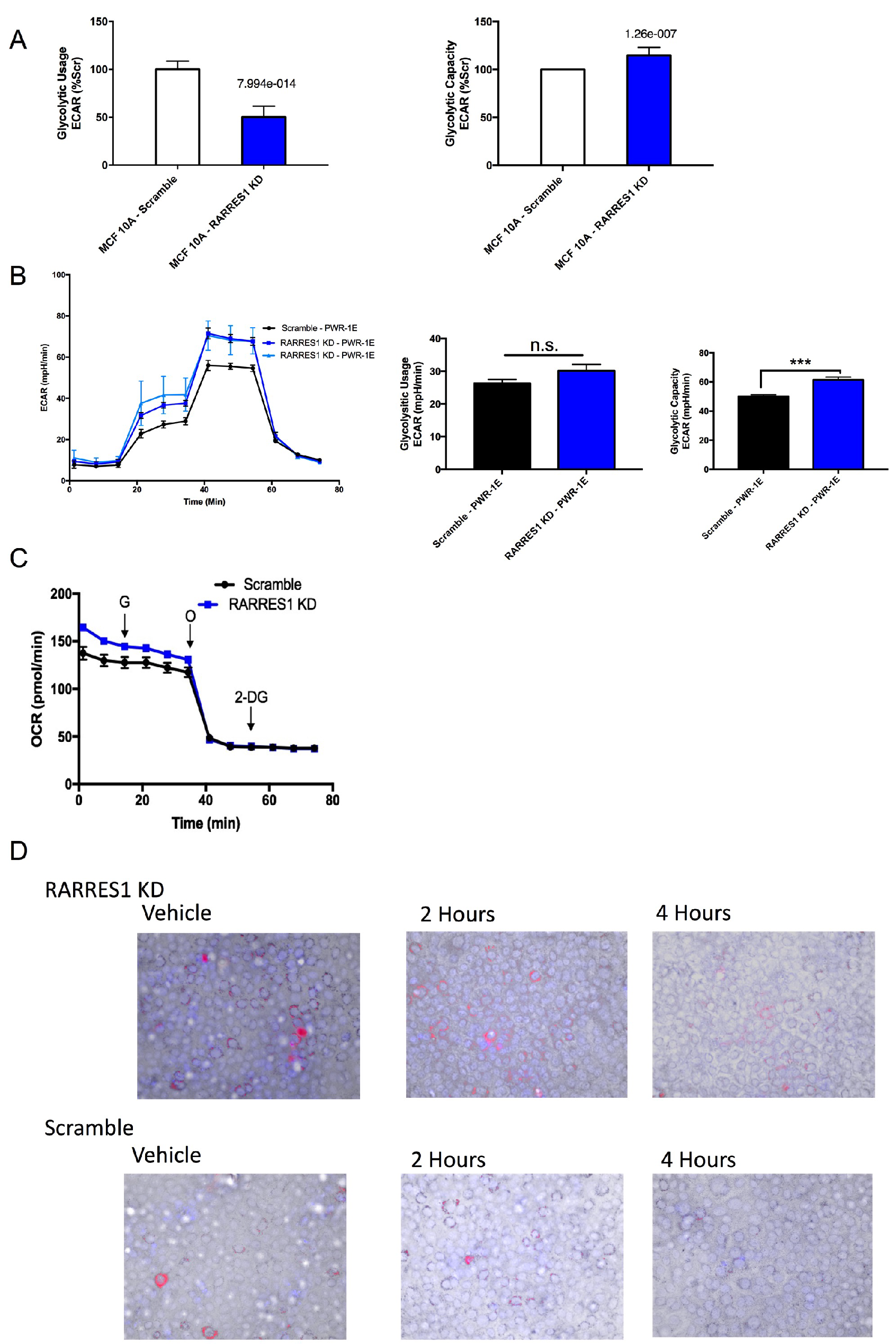

Supplement: S6 Fig — (A) Glycolytic usage and capacity was quantified in transient RARRES1 knockdown in MCF 10A cells by using the Glycolysis Stress Test. (B) Oxygen consumption rate measurement was assessed after glucose injection in the Seahorse XF Flux machine. (C) Transient glycolytic activity was assessed in PWR-1E cells with transient RARRES1 knockdown by using the Seahorse Glycolysis Stress Test. Glycolytic usage and capacity was quantified.(D) RARRES1-siRNA or scrambled siRNA transfected cells were treated with vehicle (EtOH), or 40 μM C75 for 2 hours or 4 hours. Cells were stained with Oil Red O and DAPI. (TIFF) [file pone.0208756.s006.tiff]

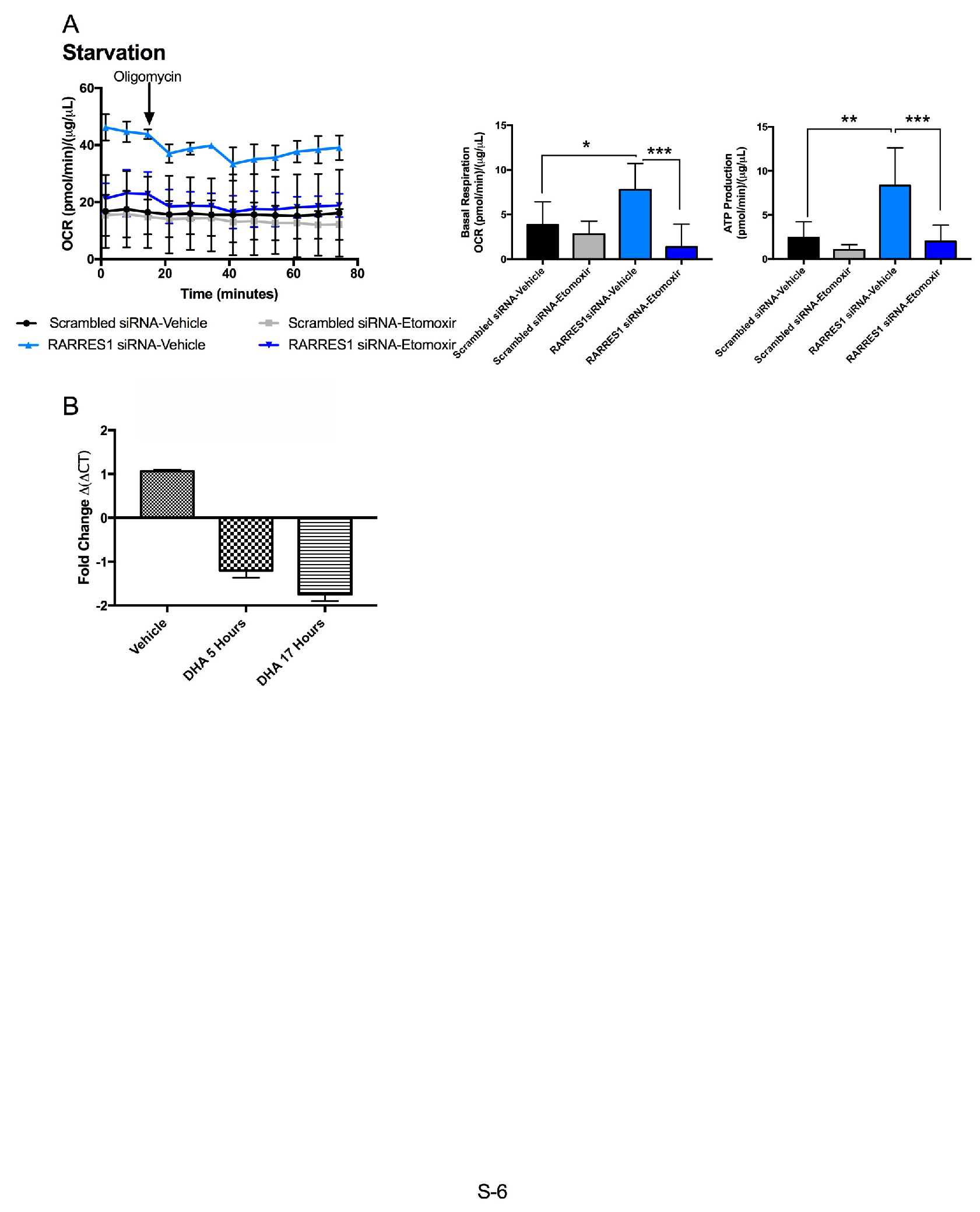

Supplement: S7 Fig — (A) PWR-1E cells were starved (1% serum and 0.5 mM glucose) and treated with etomoxir to measure fatty acid oxidation rate dependent on endogenous fatty acids. Scrambled siRNA and transient RARRES1 siRNA were measured and compared. Basal respiration and ATP production were quantified. (B) PWR-1E cells were treated with DHA for 5 hours and 17 hours. qPCR was run to assess RARRES1 expression. 18S gene was used as the endogenous control. (TIFF) [file pone.0208756.s007.tiff]

S8 Figure

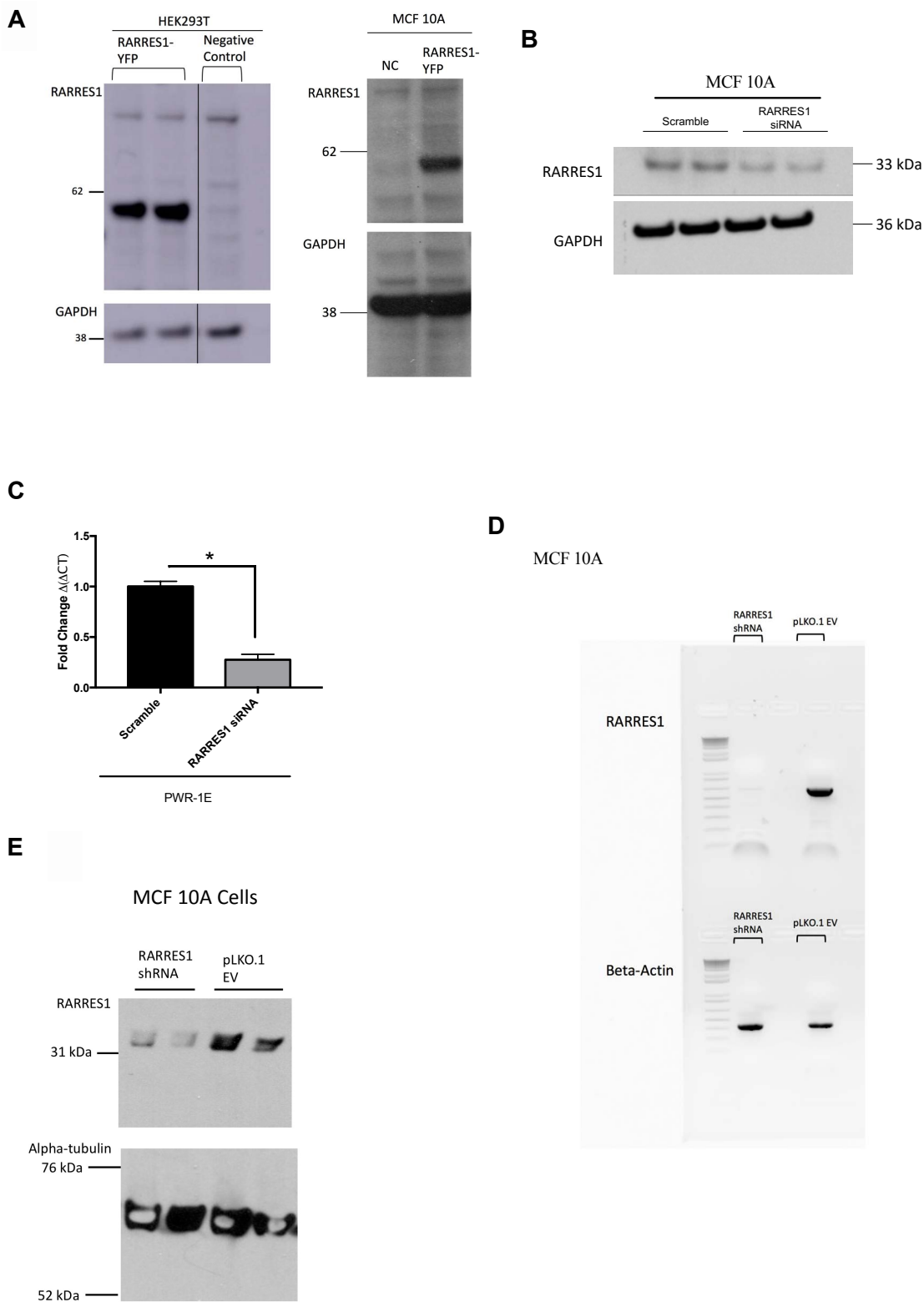

Supplement: S8 Fig — (a) RARRES1-YFP (expected band ~ 60 kDa) expression was validated in HEK 293 T cells. Tubulin or GAPDH was used as a loading control. The image was cropped, and lanes were juxtaposed; black line is drawn to describe the boundary. The full-length blot is presented in S9A Fig. RARRES1-YFP overexpression was also confirmed in MCF 10A cells. The full-length blot is presented in S9B Fig. (b) Western blot was done to confirm the transient RARRES1 knockdown efficiency in MCF 10A cells. The full-length blot is presented in S9E Fig. (C) qPCR was run to validate RARRES1 knockdown in PWR-1E cells. Transcript levels were normalized to the level of 18S. RARRES1 protein expression was confirmed in our previous study[33]. (D) Stable RARRES1 knockdown in MCF 10A cells were validated through RT-PCR. Beta-actin was used as a loading control. Full-length gel is presented in S9C Fig. (E) Stable RARRES1 knockdown was also confirmed through western blot. Alpha-tubulin was used as a loading control. Full-length blots are presented in S9D Fig. (PDF) [file pone.0208756.s008.pdf]

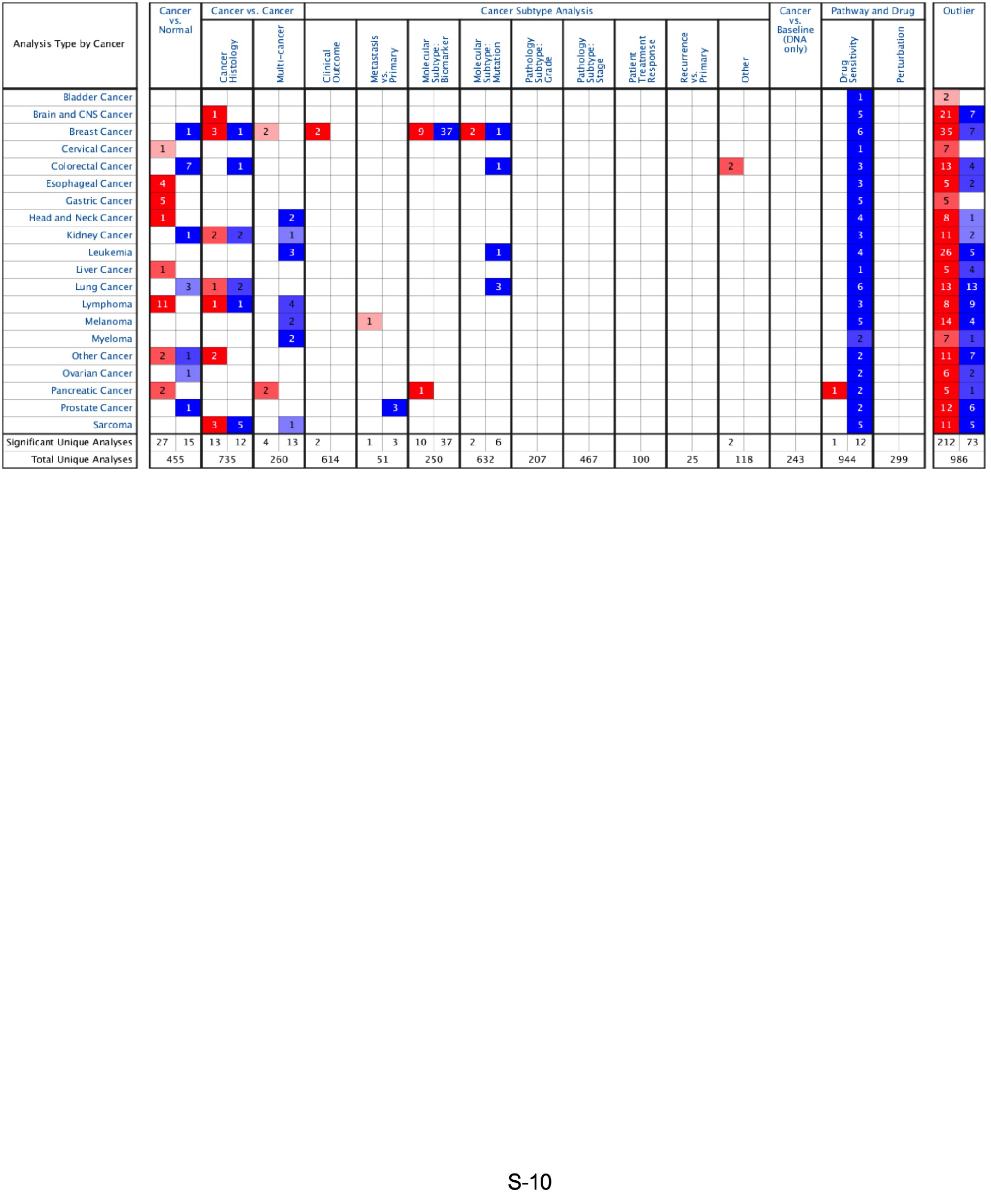

Supplement: S1 Table — The Oncomine database was queried for RARRES1 expression in the available datasets based on the following criteria: cancer type, cancer versus normal, cancer versus cancer, cancer subtype, cancer versus baseline, pathway and drug and outlier analyses. The 'red' cells represent RARRES1 overexpression and the 'blue’ cells represent RARRES1 underexpression. The levels of expression are based on the gene rank percentile. This disease summary was performed using a criterion of a 2-fold change for RARRES1 expression and a p-value of 1E-4. (Oncomine platform (C). (TIFF) [file pone.0208756.s010.tiff]

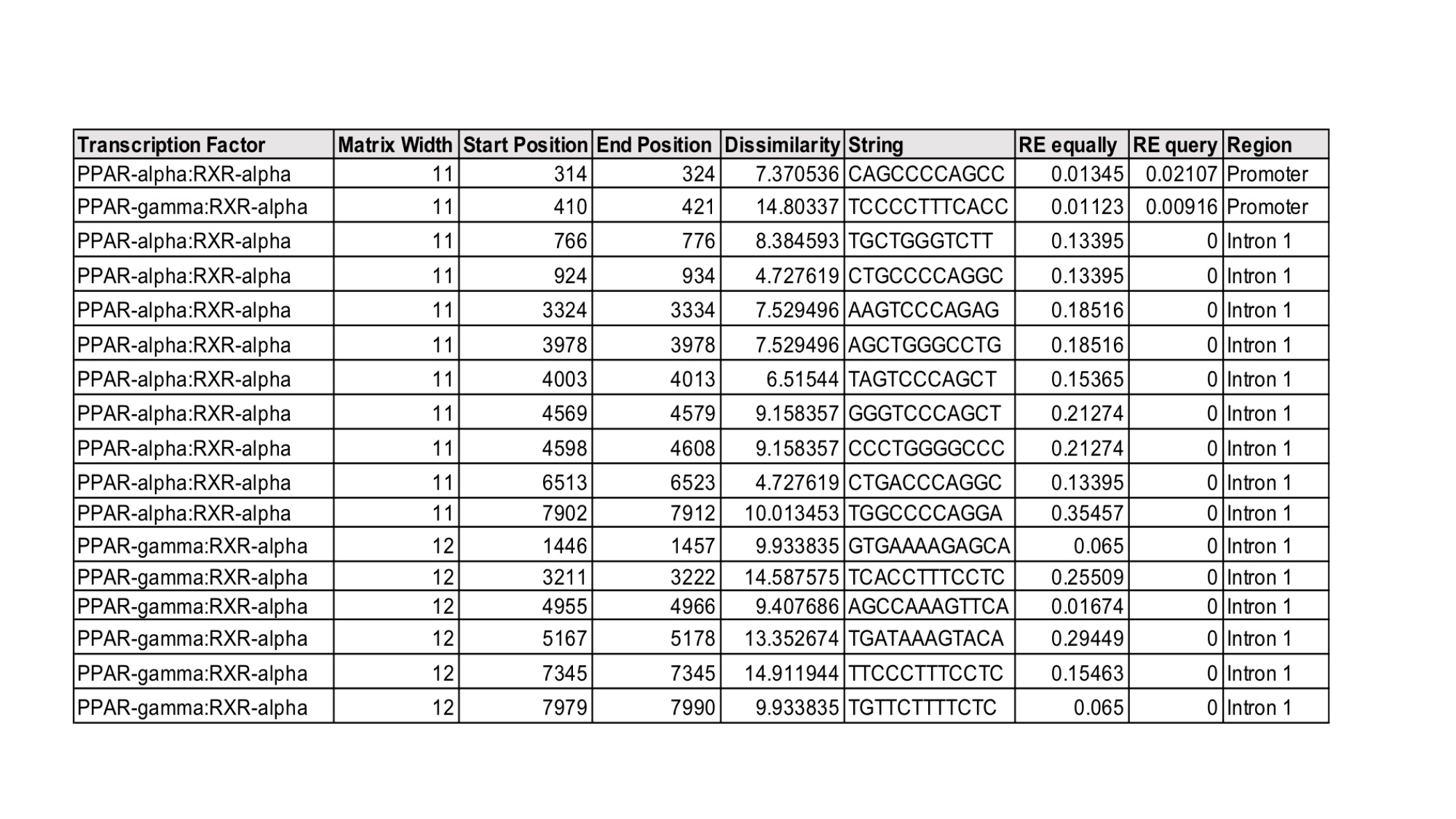

Supplement: S2 Table — In silico identification of PPAR-alpha and PPAR-gamma binding sites and within human RARRES1 gene promoter and intron 1 (non-coding regions). Raw data are shown for PPAR-alpha and PPAR-gamma binding sites in RARRES1 non-coding regions. Numbering is based on the transcription start site at position 1000. PROMO displays RARRES1 and PPAR-gamma and -alpha as indicated, along with calculated start and end positions of transcription factor (TF) binding sites. Dissimilarity values give the percent difference in sequence similarity between the RARRES1 sequence and the calculated transcription factor (TF) consensus matrix. RE, or random expectation, yields the probability that the TF consensus binding sequence would happen by chance, where 0.1 signifies 1 incidence in every 104 bases. Sequences for potential binding sites predicted by the software are included in the table. (TIFF) [file pone.0208756.s011.tiff]
