# Supplementary material for: Tumor suppressor RARRES1- A novel regulator of fatty acid metabolism in epithelial cells
Source: PLoS One. 2018 Dec 17;13(12):e0208756. doi: 10.1371/journal.pone.0208756 (PMC6296515; doi:10.1371/journal.pone.0208756)

# S2A Figure

Part I: MS/MS ESI-(+) Method: Metabolites Validated through HMDB, LIPID MAPS and Simlipid

Sphingosine; 300.29_6.163; M(+H)

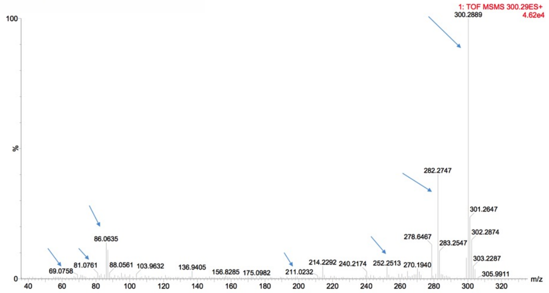


Sphinganine; 302.3024_6.432; M(+H)

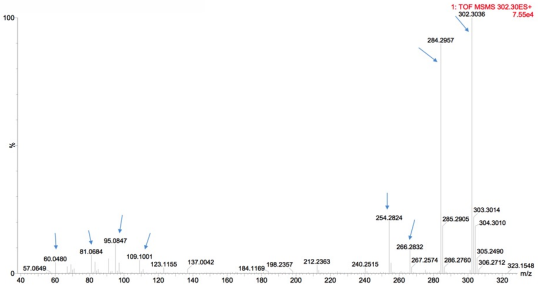


L-Palmitoylcarnitine; 400.3388_6.95; M(+$NH_4$)

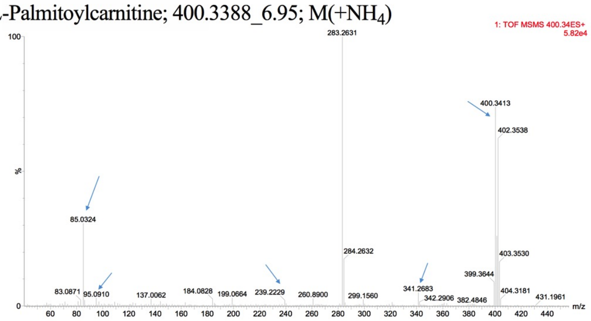


TG(60:3); 986.9042_10.64; M(+$NH_4$)

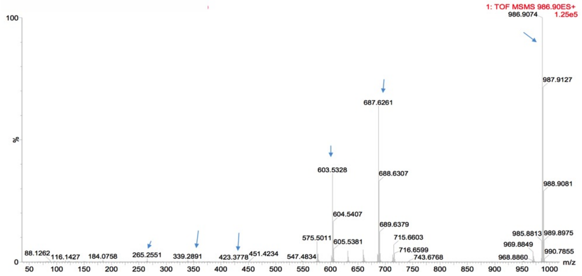


Eicostrienoic Acid; 307.263_8.29.; M(+H)

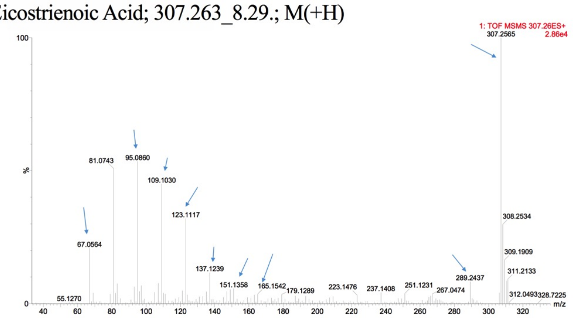


Isobutyry-L-Carnitine; 232.1538 _10.58; M(+H)

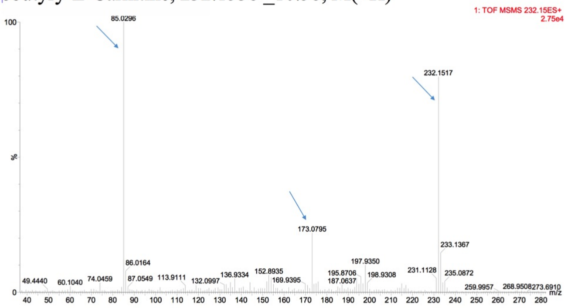


Niacinamide; 123.0556_0.425.; M(+H)

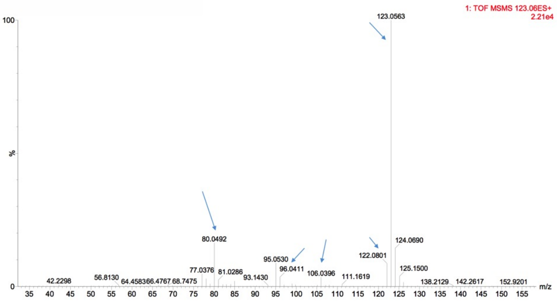

Part II**:** MS/MS ESI**-(-)** Method**:** Metabolites Validated through HMDB and LIPID MAPS**.**

PI(34:1); 835.5335_11.92; M(-H)

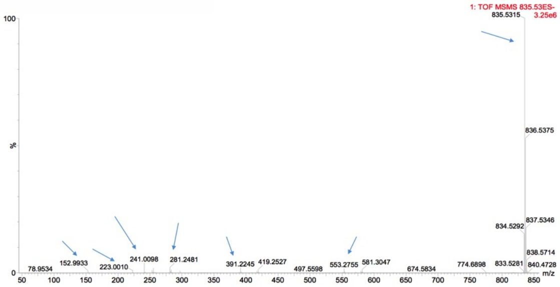

# S2B Figure

MS$^{E}$ ESI-(-) Method: Metabolites Validated through Simlipid

PE(P-16:0) ; 436.2829_6.86; M(-H)

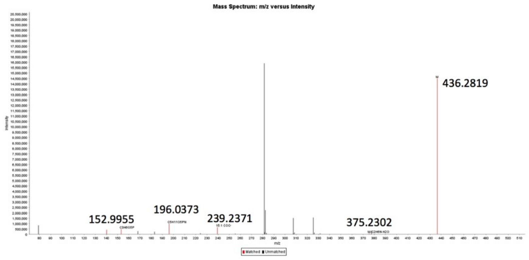


PE(34:4) ; 710.4805_9.68; M(-H)

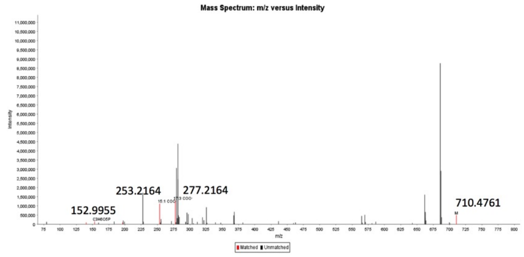


PS(38:5); 808.5077_9.92; M(-H)

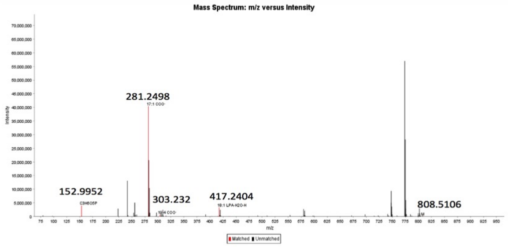


PI(34:2); 833.5178_11.93; M(-H)

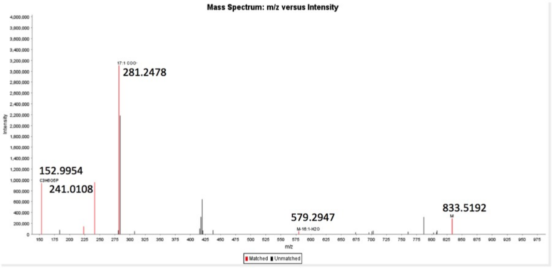


PI(34:1); 835.5335_11.92; M(-H)

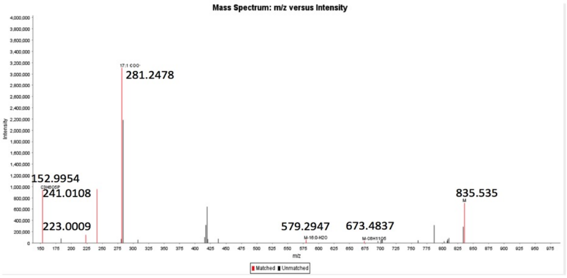


PI(36:4); 857.5206_9.91; M(-H)

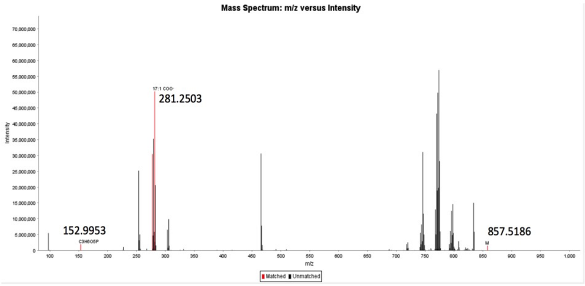


PS(44:5); 892.5989_10.33; M(-H)

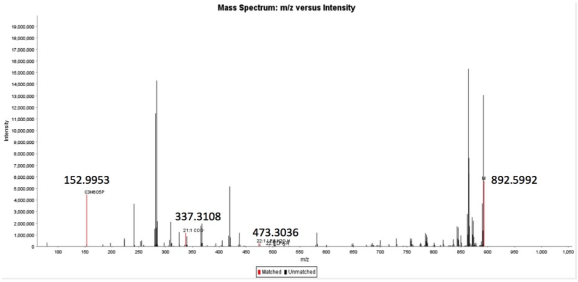

## Part I: MS$^E$ ESI-(+) Method: Metabolites Validated through SimLipid

24,25-Epoxy-cholesterol; 401.3414_9.00; M(+H)

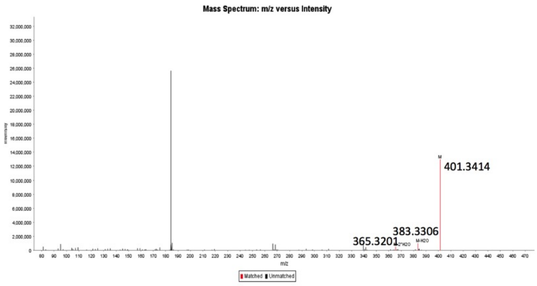


PC(O-14:0); 468.3085_5.93_; M(+H)

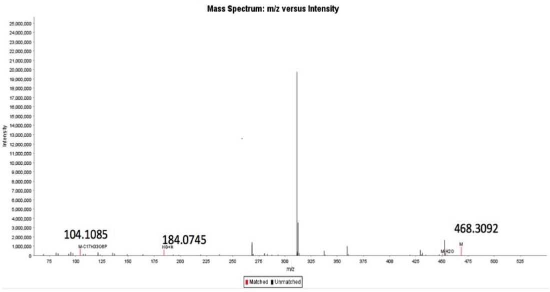


LysoPC(16:1); 494.3243_6.11; M(+H)

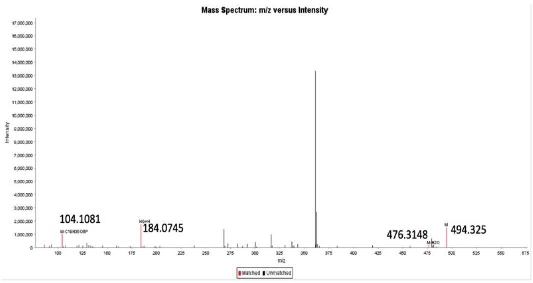


LysoPC(16:0); 496.3398_6.66; M(+H)

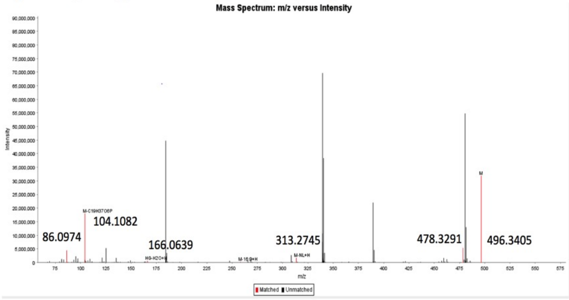


TG(54:5); 881.7565_10.4; M(+H)

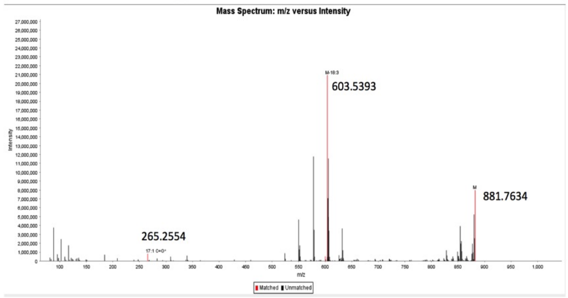


PC(O-18:1); 522.3561_5.93; M(+H)

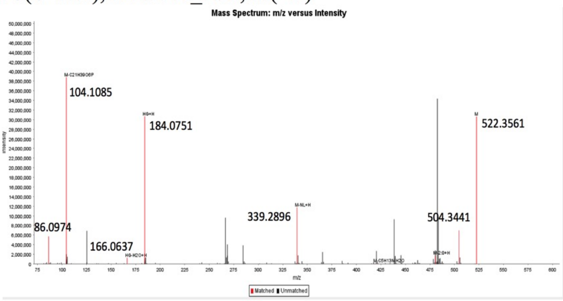


TG(62:6); 991.8662_10.6; M(+H)

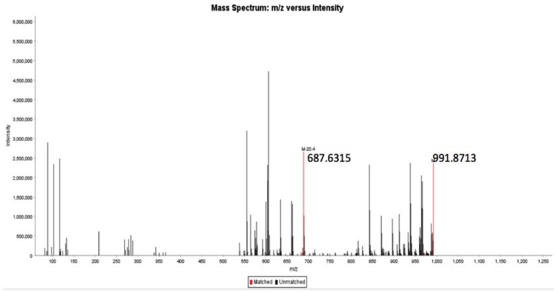

## Part II: $MS^E$ ESI-(+) Method: Metabolites Validated through SimLipid

PE(P-36:2); 728.5587_10.01; M(+H)

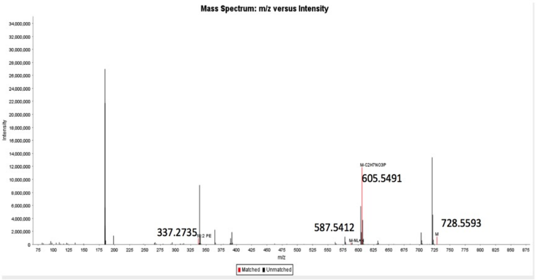


TG(52:5); 853.7343_10.4; M(+H)

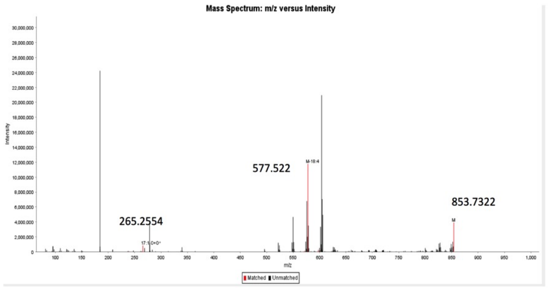


PE(20:2); 528.3065_6.4; M(+H)

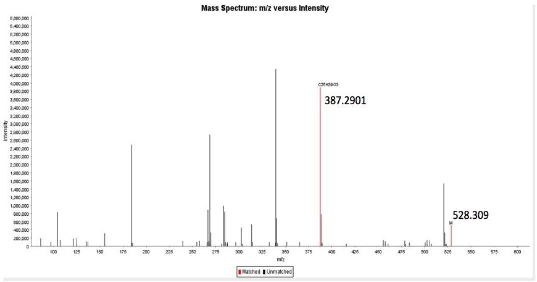


1-Palmitoyl-GPI; 595_2.793; M(+H)

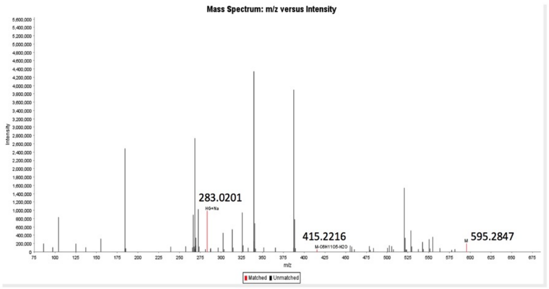

Supplement: S2 Fig — Above each graph, the lipid classification (name), mass/charge (m/z), retention time (RT), and type of adduct (M±ion), depicted as name; m/z_RT; M±ion; are included. Only metabolites with significant changes, in terms of scramble vs. RARRES1 knockdown, were validated. (A) The identity of the metabolite was validated using tandem mass spectrometry. The daughter and parent ions for the metabolites were matched with the MS/MS spectra available in HMDB, SimLipid software V6.01 (Premier Biosoft, Palo Alto, CA, USA) and LIPID MAPS [17–19]. (B) Additional validations for lipids were done through SimLipid software using MSE data. (PDF) [file pone.0208756.s002.pdf]
