# Supplementary material for: Tumor suppressor RARRES1- A novel regulator of fatty acid metabolism in epithelial cells
Source: PLoS One. 2018 Dec 17;13(12):e0208756. doi: 10.1371/journal.pone.0208756 (PMC6296515; doi:10.1371/journal.pone.0208756)

S9 Figure

A

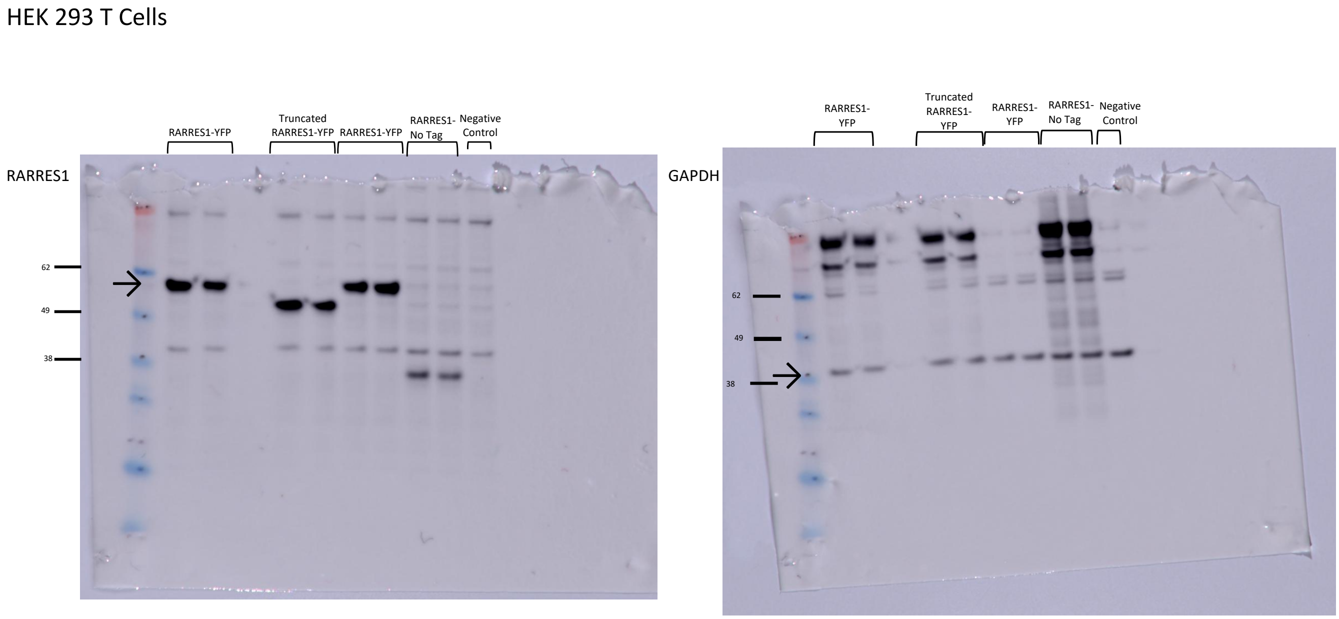

B

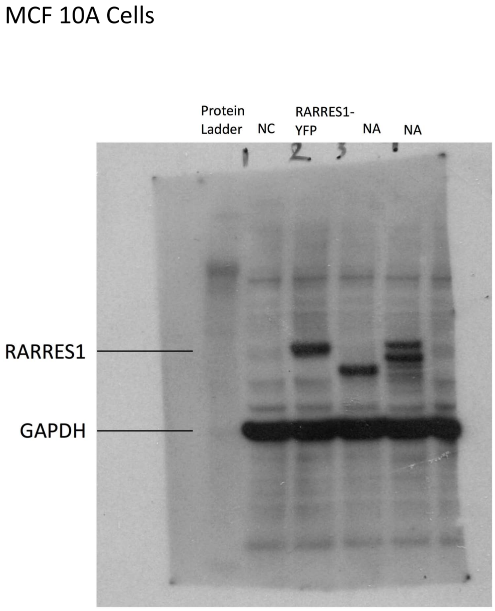

C

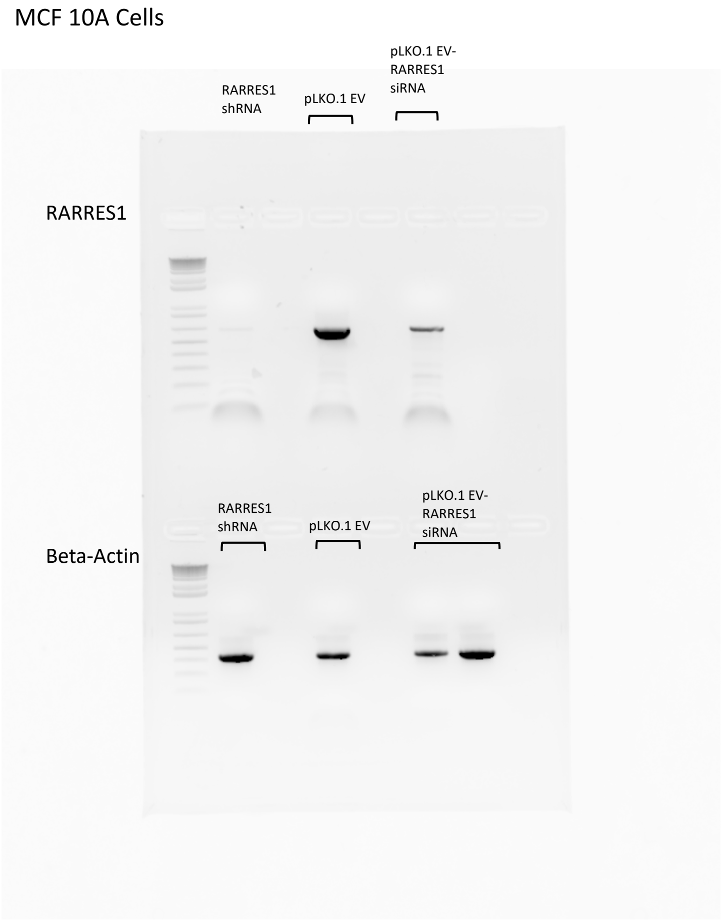

D MCF 10A

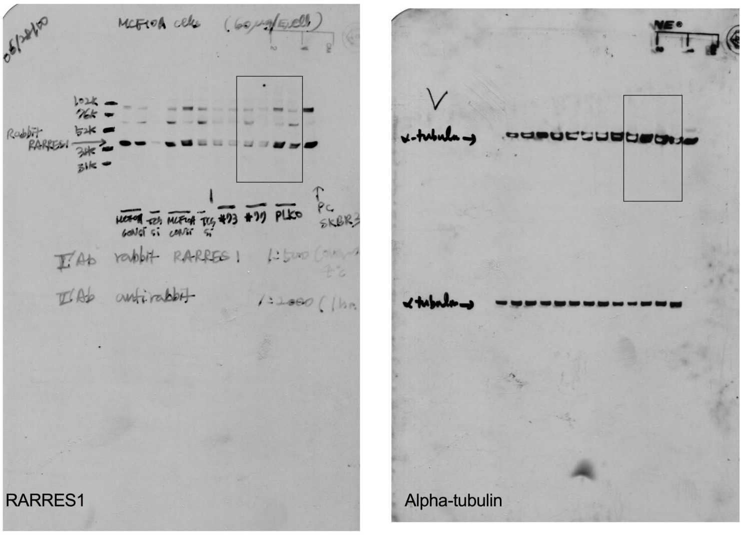

E

MCF 10A

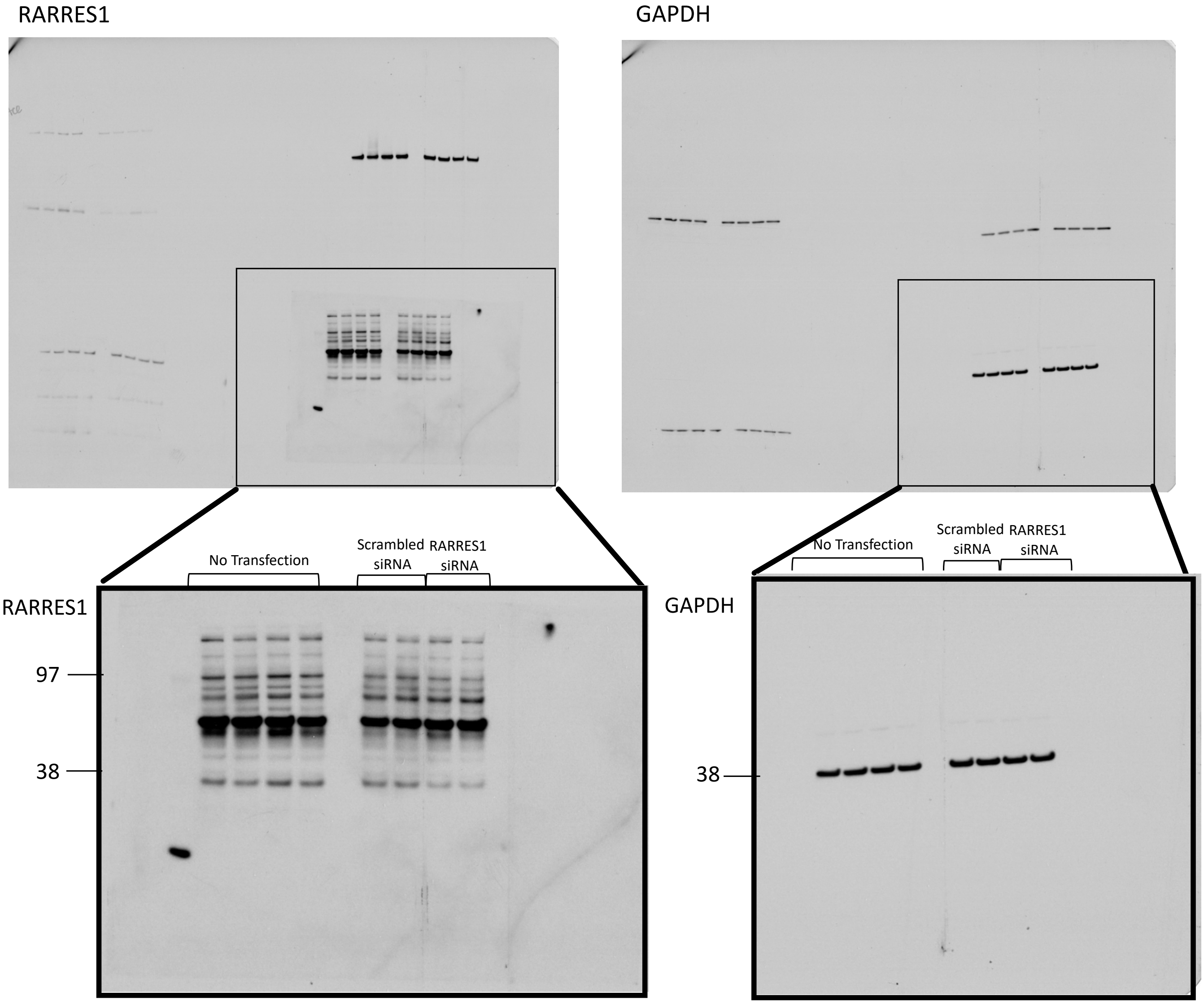

F

RARRES1

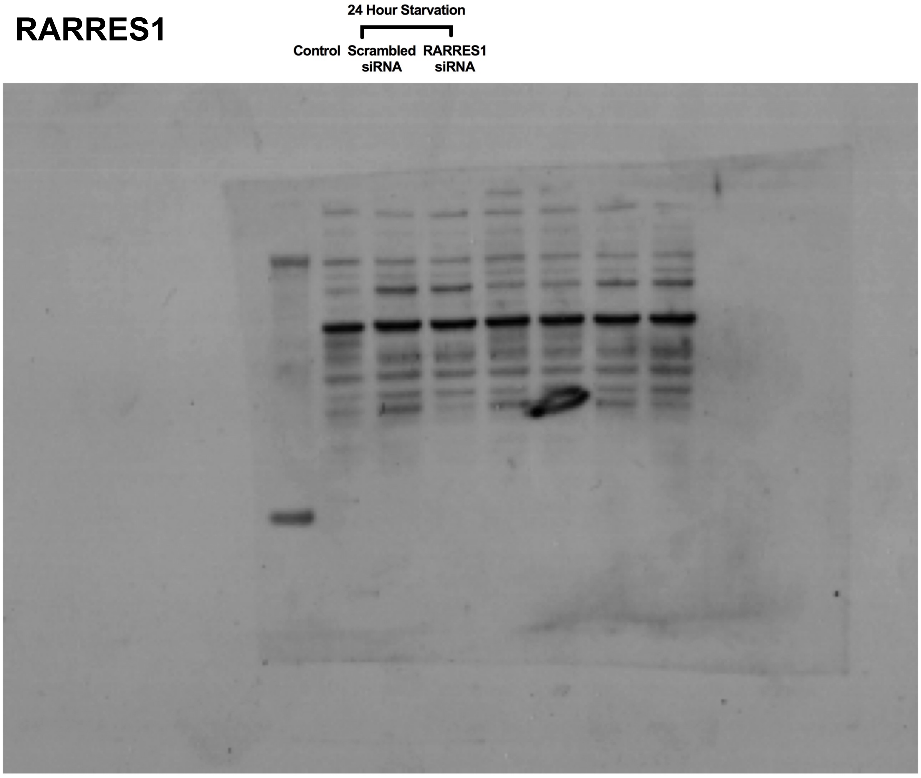

Alpha-Tubulin

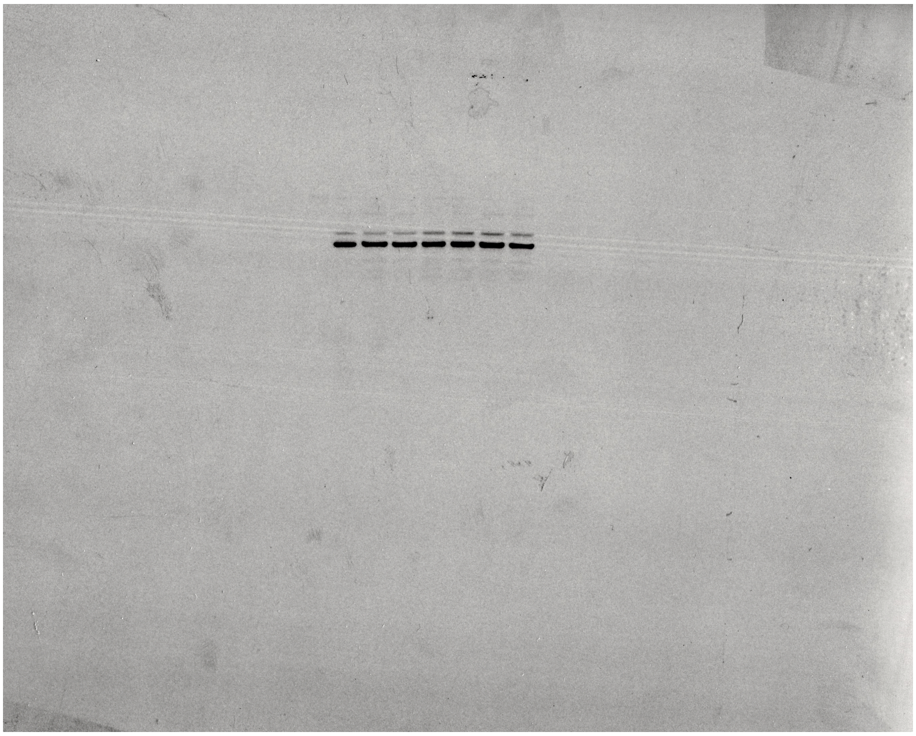

Supplement: S9 Fig — (A) RARRES1-YFP overexpression confirmation. We included transfection of truncated RARRES1-YFP and RARRES1 overexpression with no tag in order to assess the efficiency of transfection and validate that the full-length RARRES1 is being expressed. (B) Full-length blot of RARES1-YFP overexpression in MCF 10A cells is represented. NA (not applicable) was labeled on lanes that were irrelevant to the study. (C) RARRES1 knockdown efficiency was assessed. The full-length film with four different blots (examining different proteins (not relevant to this study)) is included. And the relevant blot was highlighted with black boundaries. Control cells with no transfection and scrambled siRNA and RARRES1 siRNA transfected MCF 10A cells were assessed. GAPDH was selected as a loading control. (D) Full-length blots of RARRES1 stable knockdown western blots are pictured. The left blot is probed for RARRES1 while the blot on the right is probed for alpha tubulin. The lanes that are relevant to this study are highlighted in black borders. (E) Full length gel of the stable RARRES1 knockdown MCF 10A cells. Empty vector was also transfected with RARRES1 siRNA to ensure the band is RARRES1-specific. Beta-actin was used as a loading control. Note: The third sample had to be loaded to a new lane (4th band) due to technical issues that occurred while loading sample the first time (3rd band). (F) Full length blot of the effects of serum starvation on RARRES1 western blots are pictured. The top blot is probed for RARRES1 while the blot on the bottom is probed for alpha tubulin. (PDF) [file pone.0208756.s009.pdf]
